# RGBChem: Image-Like Representation of Chemical Compounds for Property Prediction

**DOI:** 10.1021/acs.jctc.5c00291

**Published:** 2025-05-12

**Authors:** Rafał Stottko, Radosław Michalski, Bartłomiej M. Szyja

**Affiliations:** † Institute of Advanced Materials, Faculty of Chemistry, Wrocław University of Science and Technology, Gdańska 7/9, Wrocław 50-344, Poland; ‡ Department of Artificial Intelligence, Wrocław University of Science and Technology, Wyb. Wyspiańskiego 27, Wrocław 50-370, Poland

## Abstract

In this work, we introduce RGBChem, a novel approach for converting chemical compounds into image representations, which are subsequently used to train a convolutional neural network (CNN) to predict the HOMO–LUMO gap for compounds from the QM9 database. By modifying the arbitrary order of atoms present in .xyz files used to generate these images, it has been demonstrated that expanding the initial training set size can be achieved by creating multiple unique images (data points) from a single molecule. This study shows that the presented approach leads to a statistically significant improvement in model accuracy, highlighting RGBChem as a powerful approach for leveraging machine learning (ML) in scenarios where the available data set is too small to apply ML methods effectively.

## Graphical abstract

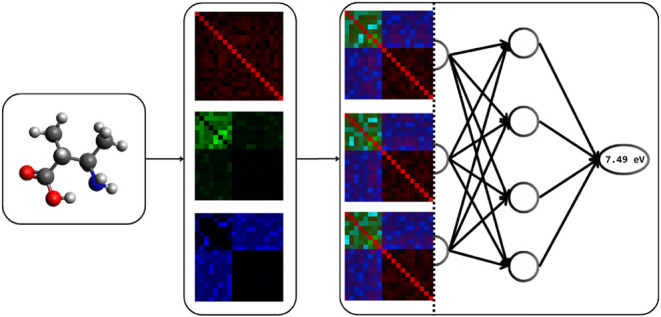

## 1 Introduction

Understanding chemical phenomena relies nowadays on computational modeling.^1,2^ However, despite continuous developments in available computational resources, many chemical processes remain difficult or impossible to study due to their complexity or the required accuracy of the simulation. Among the solutions to this problem is the reductionist approach, which uses techniques that require far fewer resources, such as QM/MM. It splits the investigated system into two subsystems described with more and less accurate methods, respectively.^3,4^ Another approach is the density functional based tight binding, which parametrizes certain energy terms.^5^ Alternatively, the golden standard DFT method can be used if the size of the investigated model is reduced, neglecting the environment of the reactive site.^2^

Artificial intelligence (AI) methods in chemistry offer alternative solutions. The foundations of deep learning date back to 1943, when McCulloch and Pitts^6^ introduced a mathematical model of a neuron. Initially, the idea was met with skepticism, as single-layer networks could not solve basic problems like the XOR gate.^7^ Due to computational limitations, deeper networks—which could address such problems—in general, remained underexplored for years.

Within the past decade, computational power has increased to the point where AI tools can easily find their way into chemistry, mostly due to the widespread adoption of specialized computational units. The research in this domain concentrates mostly on Machine-Learned Potentials (MLPs), which offer the possibility to solve computationally challenging problems that were not accessible before either by classical force fields or by DFT.^8–11^

According to Ananikov^12^ AI greatly impacts several other fields of chemistry such as Drug Discovery, Predictive Toxicology, Digital Materials Design, and Materials Informatics—to name just a few. In the domain of chemical property prediction, researchers used methods from the classical machine learning repertoire, e.g., Kernel Ridge Regression, ElasticNet, Random Forest, and other Ensemble Methods.^13–16^ Less frequently Convolutional Neural Networks (CNNs) have been used.^13,17^ Recently, two primary approaches have started to gain traction, with their applications becoming increasingly widespread: leveraging whether Graph Neural Networks (GNNs) or Graph Convolutional Networks (GCNs)^16,18^−,^19,20^ or—by using SMILES^21^ or SELFIES^22^ representation—treating chemical compounds as sequences and applying to them methodologies from the Natural Language Processing (NLP) domain.^23–25^

In this work, we introduce RGBChem, a proof-of-concept approach for representing the information embedded in chemical compounds through image-based encoding. These are treated in the next step as training data for a convolutional neural network and can be successfully used to predict the properties of chemical compounds. RGBChem enables the generation of multiple data points from a single chemical compound, which facilitates the training of models in cases where the training data set contains a limited number of chemical compounds. This approach promotes the development of more specialized models, for which, under other conditions, the size of the database would effectively prevent efficient training. The name of the method, RGBChem, is inspired by the RGB color model. These three color spectra encode up to six distinct aspects of a compound into three matrices, each corresponding to the red, green, or blue spectrum.

We tested our approach to predict the HOMO–LUMO gap energy, achieving an accuracy of 272 meV in the best case. To train our models, we utilized compounds from the QM9 ^26^ data set, which is the standard data set for proof-of-concept models in quantum chemistry prediction tasks.

## 2 Related Work

CNNs and GNNs share several similarities, as information can be effectively represented using both matrices and graphs. A good example is the geometry of chemical compounds expressed as the distances between atoms. On the one hand, they can be represented as a distance matrix, while on the other, they can take the form of a graph, where each vertex represents an atom and each edge length corresponds to the atomic distance.

The primary distinction lies in how neural networks learn in each instance. CNNs rely on local patterns, relations between spatially closed information encoded in the form of pixels on images, and thus relations that depend on specific ways of presenting data in visual form.^27^ In contrast, GNNs, especially transformers,^28^ capture interactions at different scales and focus more on global patterns. It is also worth mentioning that the input data in GNNs are order-independent.

This leads us to the conclusion that graph-based representations of chemical systems is more intuitive and may lead to better results based on current knowledge^29^ and technical background. The best-known architecture based on neural networks with continuous filtering, SchNet^30^ achieves a HOMO–LUMO gap prediction accuracy of 63 meV on the QM9 set, while to the best of our knowledge, the state-of-the-art based on the multilevel attention mechanism^31^ achieves an accuracy of 32 meV. This, however, comes at the cost of more complex training procedure, and the requirement for more training data.

Numerous diverse and innovative approaches have been explored for predicting the HOMO–LUMO gap. The accuracies of these approaches are presented in Table 1.

**Table 1:** Results from Other Research Teams on Predicting the HOMO–LUMO Gap Energy (eV)

ref.	ML Method	Train set (size)	Test set (size)	Mean Abs. Error bandgap (eV)
^31^	DeepMoleNet	QM9 (110,000)	QM9 (9428)	0.032
 ^33^	Cormorant	QM9 (100,000)	QM9 (16,946)	0.061
 ^30,31^ ^a^	SchNet	QM9 (110,000)	QM9 (9428)	0.063
 ^18^	MEGNET	QM9 (104,369)	QM9 (13,046)	0.066
 ^34^	enn–s2s	QM9 (110,462)	QM9 (10,000)	0.069
 ^16^	GNN	QM9 (118,000)	QM9 (13,100)	0.087
 ^35^	Neural Network	QM9 (107,108)	QM9 (13,885)	0.091
 ^13^	CNN	custom (21,012)	custom (2627)	0.273
 ^13^	Random Forest	custom (21,012)	custom (2627)	0.493, 0.532, 0.536^b^
 ^16^	ML	QM9 (118,000)	QM9 (13,100)	0.441, 0.209, 0.126^c^
	RGBChem	QM9 (108,000)	QM9 (13,885)	0.272
	RGBChem	1/4 of QM9 (27,000)	QM9 (106,885)	0.317
	RGBChem	1/6 of QM9 (18,000)	QM9 (115,885)	0.369

a The approach was derived from ref 30 and the results were based on ref 31.

b In the cited work, three different RF approaches have been applied.

c Mean accuracies for the elastic net, random forest, and kernel ridge regression, respectively.

We identified models that transform chemical information into a form suitable for CNNs. While they share some similarities with our approach, they remain fundamentally different. For example, Casey et al.^13^ used a 3D electronic structure and 4D electrostatic potential and then employed a CNN to jointly train a model for predicting eight different properties. Another approach that shares some aspects with RGBChem was developed by Krüger et al.^17^ They used the SMILES representation and applied a one-hot encoding technique to create a 2D representation suitable for training a CNN to predict the one-electron reduction potentials of compounds.

The challenge in limited data availability training has been addressed by Heinen et al.^32^ Their approach focused on fine-tuning the loss function, rather than expanding the training data set. Still, they observed that the mean absolute error of the method strongly depends on the size of the training set, with larger data sets leading to improved model performance.

## 3 Methods

### 3.1 Data Set

For the purpose of training, we have used the QM9 database,^26^ which is among the most widely used chemical compound databases by researchers building models to predict quantum properties. It consists of 133,885 compounds containing up to 9 heavy (non-hydrogen) atoms. Besides the whole QM9 database, we considered the subsets of QM9 created by a random extraction of 1/n of compounds from the QM9 data set (e.g., 1/4, 1/6). These data sets will be named QM9_*N*, where *N* is the denominator of the fraction (e.g., QM9_2 is the randomly selected half of QM9 compounds, and QM9_6 data set is 1/6 of all QM9 compounds). Subsets of QM9 were created to model a scenario in which we deal with a deficiency of training data points.

There is an ongoing discussion about the completeness QM9 data set in terms of its limited composition (restricted to C, H, O, N, and F atoms) and its narrow range of functional groups. This issue was highlighted by Glavatskikh et al.^36^ who compared the accuracy and generalizability of models trained on QM9 with those trained on their own PC9 data set. Their study revealed a noticeable drop in model accuracy when trained on QM9 and validated on PC9. Despite this, we chose to primarily use the QM9 data set due to the vast number of studies that have also relied on it, allowing for the comparison of our RGBChem method with other works.

### 3.2 Neural Network Architectures

We evaluated both predefined architectures for computer vision tasks and experimented with proposing our own. Among the tested, well-known solutions, we considered architectures from the ResNet family^37^ (ResNet18, ResNet34, ResNet50), DenseNet121 ^38^ SqueezeNet1_1, SqueezeNet1_0,^39^ VGG16_bn and VGG19_bn.^40^ Additionally, a custom family of CNN architectures was also developed (SnCNN). All models originally designed for classification tasks had their final layer removed, allowing us to repurpose them for a regression task.

(1) ResNet: deep learning architectures with many layers (18, 34, and 50 for ResNet18, ResNet34, ResNet50 respectively) which solves the problem of vanishing gradients by applying so-called ″skip-connections″. These architectures have 11.7, 21.8, and 25.6 million parameters, respectively.
(2) DenseNet: A deep learning architecture that improves information flow by utilizing dense connections, where each layer receives input from all previous layers. This design helps mitigate the vanishing gradient problem and promotes feature reuse. DenseNet-121, which we tested in this work, has 8 million parameters—a surprisingly small number considering the size of the model.
(3) SqueezeNet: light in terms of the number of parameters, deep learning architecture designed to achieve AlexNet-level accuracy with significantly fewer parameters. It utilizes ″fire modules″ that combine 1 × 1 and 3 × 3 convolutions to reduce the number of parameters while retaining expressive power. SqueezeNet1_0 is the original version, while SqueezeNet1_1 introduces slight modifications to reduce the model size and improve speed. Both of these architectures have around 1.2 million parameters.
(4) VGG: A deep convolutional neural network architecture known for its simplicity and depth, where the primary building block is a stack of 3 × 3 convolutional layers followed by pooling layers. VGG16_bn has around 138 million parameters while VGG19_bn exceeds 143 million. Despite large size and memory usage VGG-like architectures are widely used for their strong performance and ability to capture hierarchical image features.
(5) SnCNN: A custom family of CNN architectures developed specifically for this study, aiming to explore even simpler alternatives than SqueezeNet. SnCNN architectures are designed with a minimalistic approach, employing a limited number of convolutional layers (one for S1CNN and two for S2CNN) followed by three fully connected layers. The S1CNN model comprises approximately 0.2 million parameters, while S2CNN—due to an optimized design—requires only about 0.1 million parameters.

When selecting architectures, the popularity of available solutions was initially prioritized. Thus, well-established and thoroughly documented architectures, known for achieving strong benchmark results, were selected. These architectures, however, might be overly complex, as they are often designed for analyzing images of significantly larger sizes. To consider this effect, we examined a wide range of architectures: from large-scale models like VGG and DenseNet, through smaller prebuilt architectures such as SqueezeNet, and finally to highly compact architectures, which we had to design ourselves which have 3 orders of magnitude fewer parameters than VGG.

### 3.3 Hyperparameters

We experimented with different momentum and learning rate values. As for the optimizer, we always chose Adam.^41^ We also implemented an early stopping mechanism with the constant delta value (Δ = 0) and varying values for the patience parameter. The overview of the workflow is shown in Figure 1.

**Figure 1:**
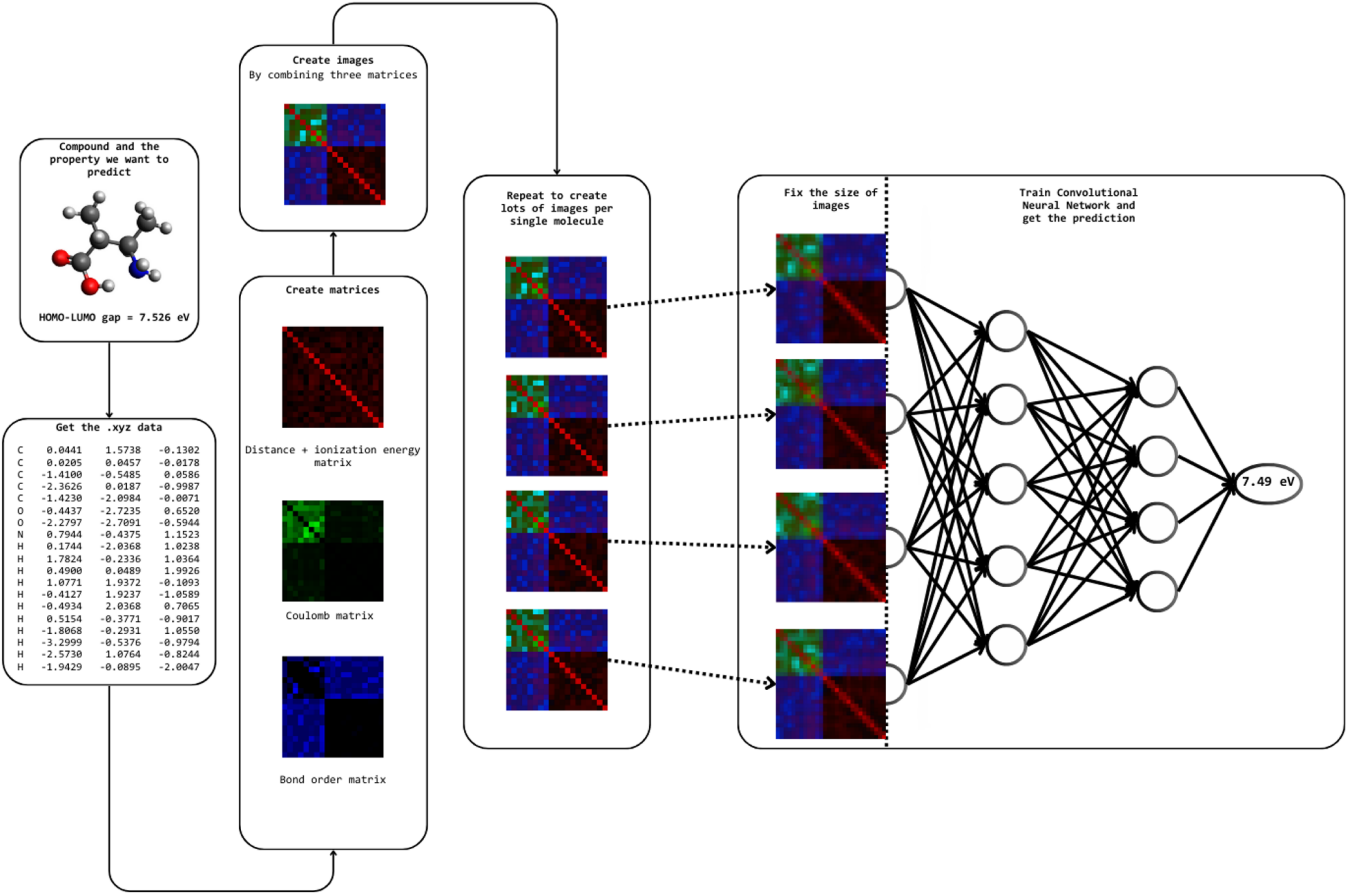
Overview of the RGBChem workflow.

### 3.4 Image Generation Procedure

The general idea behind the methodology used in the present work is to create multiple matrices (*N* + *M*) × (*N* + *M*), where *N* is the number of atoms of a given structure and *M* is the size of the margin, which is required to make the matrices uniform in size. Then, the properties relevant to the atoms are put in the matrix diagonal, and the interatomic properties are put off-diagonally. Up to six different properties are considered to generate three independent matrices, with the values normalized between 0 and 255. Then the matrices are superimposed, so each of the three matrices contains information about each cell’s red, green, and blue content, making in effect (*N + M*) × (*N + M*) × 3 size tensor.

In this way, we created a particular way of representing up to six (3 diagonal and 3 off-diagonal) chemical properties with a single image. In some specific configurations, one property that takes nonzero values in cells where *i* ≠ *j*, and another property that takes nonzero values only in cells where *i* = *j* can be included in one color space (e.g., matrix distance and ionization energy can be presented in one matrix, one color spectra). Selecting all properties on this basis, we gain a representation using an image of six chemical properties. We were then able to pass such transformed data to a convolutional neural network (CNN) and proceed with the training procedure.

#### 3.4.1 Chemical Properties Used to Generate Images

In our search for the optimal selection of properties, we distinguished properties that are characteristic of atoms, interactions between atom pairs, and both. We were particularly focused on the descriptors easily obtainable from the available chemical databases or tables (e.g., ionization potential) or possible to calculate easily (e.g., interatomic distance, bond order). Thus, we have specifically relied on nonquantum chemical parameters. Indeed, the QM9 data set—including the molecular geometries—was generated using DFT calculations, however, we emphasize our workflow does not require any additional quantum chemical calculations. For other compounds, not present in the data set, geometries can be obtained using significantly less computationally demanding methods.

(1) Properties characteristic for atoms: The methodologies applied to determine the properties of ionization energy, and atomic number were consistent across all elements. These values were sourced systematically for all atomic types from the database available at ptable.com.
(2) Interatomic distance: In *a*_*ij*_ cell the value is given by
1 $$R_{ij}=\sqrt{{\left(x_{i}-x_{j}\right)}^{2}+{\left(y_{i}-y_{j}\right)}^{2}+{\left(z_{i}-z_{j}\right)}^{2}}$$
  where the notation represents the *x*, *y*, and *z* coordinates of the atom *i* and *j*, for instance: *x*_*i*_ is the *x*-coordinate for *i*-th atom, and *z*_*j*_ is *z*-coordinate value from *j*-th atom. The distance always equals 0 when *i* = *j*. Interatomic distance is a descriptor for the atom pairs.
(3) Coulomb matrix: has been determined according to the formula:
2 $$M_{ij}^{\mathrm{Coulomb}}=\left\{ \begin{array}{l} 0.5Z_{i}^{2.4},\mathrm{for}\;i=j\\ \frac{Z_{i}Z_{j}}{R_{ij}},\mathrm{for}\;i\neq j \end{array}\right.$$
  where *Z* is the nuclear charge of a given atom and *R*_*ij*_ is the Cartesian distance between atom *i* and *j*. Coulomb matrix is a descriptor for both: atoms and atom pairs. However, in the course of testing, we noticed that in our case, the values for cells where *i* = *j* were much larger numerically than for cells where *i* ≠ *j*. Thus, we tested the use of a Coulomb matrix that had zeros on the diagonal (atom pairs’ descriptor only). To conveniently distinguish between the two types of Coulomb matrix formation, we use the notation of full Coulomb matrix which considers the nonzero values are in all matrix cells (*i* = *j* and *i* ≠ *j*) and reduced Coulomb matrix which takes nonzero values only in the off-diagonal cells (*i* ≠ *j*).
(4) Bond order: to determine the bond order we used the ReaxFF approach.^42,43^ We used parameters from reaxff_cohnsli.lib file from Gulp library^44^ by substituting −1 value for *r* ^σ^, *r* ^π^ and *r* ^*ππ*^ for O–F, F–N, and F–H interactions regardless whether they are available or not. This implies that we ignore fluorine interactions other than those with carbon since these are interactions that rarely occur in the structures we studied. Bond order is an atom-pair descriptor, and is given by a formula:
3 $$BO_{ij}^{\prime }=BO_{ij}^{\sigma }+BO_{ij}^{\pi }+BO_{ij}^{\pi \pi }$$
  where
4 $$BO_{ij}^{\sigma }=\exp\left[p_{bo1}\times {\left(\frac{r_{ij}}{r_{o}^{\sigma }}\right)}^{p_{bo2}}\right]$$
5 $$BO_{ij}^{\pi }=\exp\left[p_{bo3}\times {\left(\frac{r_{ij}}{r_{o}^{\pi }}\right)}^{p_{bo4}}\right]$$
6 $$BO_{ij}^{\pi \pi }=\exp\left[p_{bo5}\times {\left(\frac{r_{ij}}{r_{o}^{\pi \pi }}\right)}^{p_{bo6}}\right]$$

For clarity, we have predefined various algorithms for generating images, each assigned a specific letter designation (A, B, C, etc.). Table 2 outlines the definitions corresponding to each letter, while Figure 2 presents visual examples of these image types for reference.

**Table 2:** Types of Image Creation: *Italic* Font Corresponds to Properties Characteristic to Individual Atoms (Diagonal of the Matrices), **Bold** Denotes the Properties of Atoms Pair (Off-Diagonal Elements of the Matrices), and ***Italic–bold*** Font Represents Properties Characteristic to Both Diagonal and Off-Diagonal Elements

Name	red color	green color	blue color
A	**distance matrix** + *ionization*	**reduced Coulomb matrix**	**bond order**
B	**distance matrix** + *ionization*	***full Coulomb matrix***	**bond order**
C	**distance matrix**	**reduced Coulomb matrix**	**bond order**
D	**distance matrix**	**reduced Coulomb matrix**	**bond order** + *atomic number*
E	*ionization*	***full Coulomb matrix***	*atomic number*
F	**distance matrix** + *ionization*	***full Coulomb matrix***	*atomic number*

**Figure 2:**
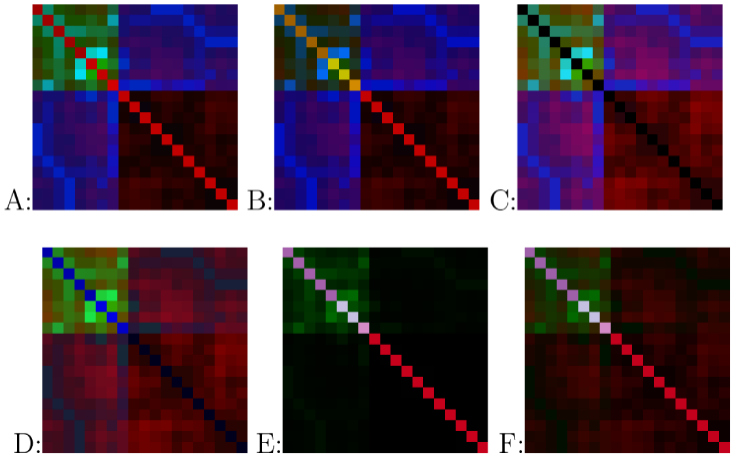
Examples of output images. From top-left to bottom-right: (A–F) image generation procedures. All images represent the same compound: 7447, from the QM9 database. Shuffling type: none.

Each type of image generation has some characteristics shared with another type, differing by a single difference within one color spectrum. This implies that two color channels remain identical between corresponding image generation types. For example, the F–B pair differs specifically in the blue spectrum due to the addition of atomic number information in F, while the red and green spectra remain unchanged. Similarly, the A–B differ in the green spectrum, where diagonal information from Coulomb matrices is added in the latter.

#### 3.4.2 Impact of Permutation Invariance

3.4.2 The chemical compounds found in the databases on which we train our models have different numbers of atoms, which creates an obvious conflict with convolutional neural networks requiring the same dimensions of the images. To investigate this effect we tested the following solutions: filling empty pixels by scaling an *N* × *N* image into defined *(N+M)* × *(N+M)* size (referred to as *resize*); filling empty pixels with either black or the average of the numerical representation of color in the RGB scale from the *N* × *N* image. For the latter two options, we also considered scenarios where the image is either randomly positioned within the (*N* + *M*) × (*N* + *M*) (referred to as *random*) or fixed to the top-left corner (referred to as *false*). For the *resize* option, the *random* and *false* positioning techniques are not applicable.

Moreover, by artificially creating order in our representation, it is necessary to use an approach that allows the elimination of variance when permuting the order of atoms in the output compound.

In order to investigate this effect we tested the following solutions:

(1) A randomization of the order of atoms each time an image is generated (i.e., *shuffling*). We distinguish between different types of shuffling:
  (a) Completely random selection (e.g., *CO* ^1^ *O* ^2^ *H* ^1^ *H* ^2^ → *O* ^2^ *CH* ^1^ *H* ^2^ *O* ^1^): *full.*
  (b) Dividing atoms into two groups: carbon, oxygen, nitrogen, and fluorine (CONF) in one group of atoms and hydrogens in the other together with random selection inside these two groups (e.g., *CO* ^1^ *O* ^2^ *H* ^1^ *H* ^2^ → *O* ^1^ *O* ^2^ *CH* ^2^ *H* ^1^): *partial.*
  (c) Random mixing in the same types of atoms (e.g., *CO* ^1^ *O* ^2^ *H* ^1^ *H* ^2^ → *CO* ^2^ *O* ^1^ *H* ^1^ *H* ^2^): *groups.*
  (d) A control sample, in which we considered models with the order of the atoms hard-defined (e.g., *CO* ^1^ *O* ^2^ *H* ^1^ *H* ^2^ → *CO* ^1^ *O* ^2^ *H* ^1^ *H* ^2^): *none.*

These techniques allowed us to introduce several possible permutation invariance types, as we investigate how these different forms impact the model’s final performance. Visual examples of these various techniques are presented in Figures 3 and 4. A detailed discussion of the impact of these techniques is provided later in the paper.

**Figure 3:**
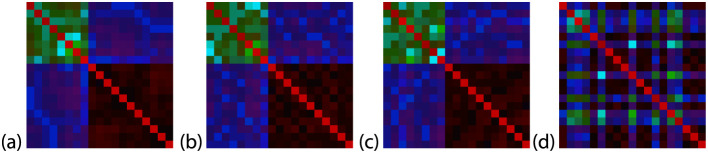
Examples of output images with different shuffling types. (a) none, (b) groups, (c) partial, and (d) full shuffling. All images represent the same compound: 7447, from the QM9 database. Image generation type: A.

**Figure 4:**
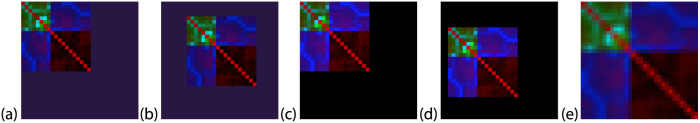
Examples of output images, illustrating the effect of unifying image sizes: (a) no rescaling, background filled with average value with position fixed to top-left corner; (b) no rescaling, background filled with average value, and random offset within the canvas; (c) background filled with black, with position fixed to the top-left corner; (d) black filling with random offset within the canvas; and (e) bilinear resize technique (in this scenario random offset parameter is not applicable), are presented. All images represent the same compound: 7447, from the QM9 database. All presented images have a resolution of 32 × 32 pixels; however, the image size is a variable parameter that will be discussed further.

Additionally, randomly selecting the order of atoms each time enables multiple representations for a single molecule, significantly increasing the amount of training data available (see Figure 5). This provides a key advantage over other models, where only one data point per molecule is used, allowing for more effective model training. Importantly, this technique preserves the unique structural characteristics of molecules, ensuring for example, that constitutional isomers remain distinguishable despite the variability in representation. Furthermore, by generating multiple representations of a single molecule, we substantially reduce the influence of data preparation on the final prediction outcomes, which is highly desirable. Our goal is to convey the underlying chemical information on the systems, rather than the specific notation used to represent compounds.

**Figure 5:**
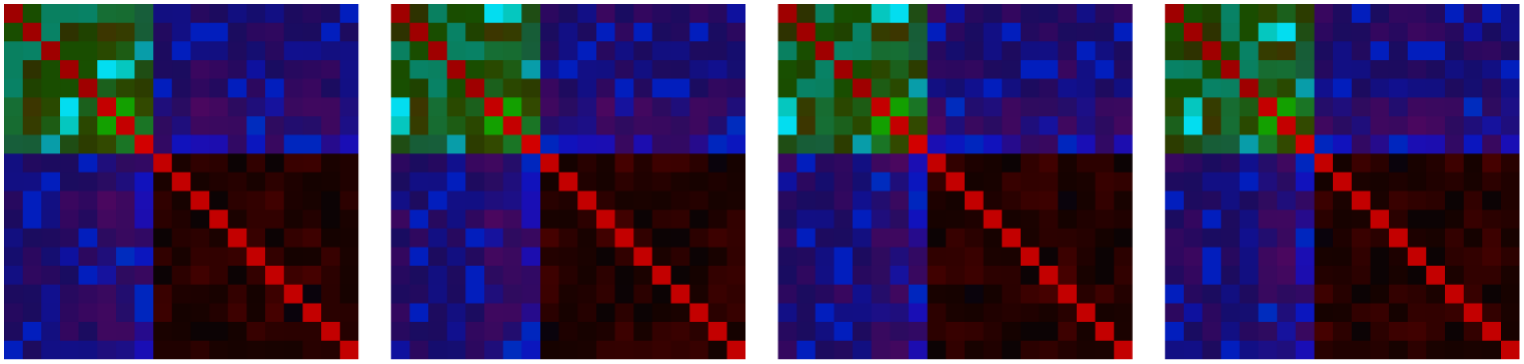
An example of multiple images based on a single compound. All of these images are generated using the same technique (shuffle: groups, image type generation: A) and the same compound (7447 from the QM9 database). This approach is applicable to all shuffling types, except for the ″none″ shuffling type.

## 4 Workflow

We consider the following options for different parameters:

(1) Databases: QM9_1, QM9_2, QM9_3, QM9_4, QM9_6.
(2) Number of images per molecule or a number of data points in the training set as described in Table 3.
(3) Type of image generation: A, B, C, D, E, F (see Table 2)
(4) Batch size: 16–128 (iteration by 4).
(5) Shuffle type: none, partial, groups, full (see Figure 3).
(6) Margins type: resize, average value, black, average+random offset, black + random offset (see Figure 4).
(7) Size of images used to training: 32 × 32, 36 × 36, 40 × 40, 44 × 44, 48 × 48
(8) Neural Network Architecture: ResNet18 (r18), ResNet34 (r34), ResNet50 (r50), VGG16_bn (vgg16), VGG19_bn (vgg19), DenseNet121 (dn121), SqueezeNet1_0 (sqn10), SqueezenNet1_1 (sqn11), S1CNN (S1), S2CNN (S2).
(9) Patience: 16–44 epochs (iteration by 4).
(10) Value which model predicts: HOMO–LUMO gap.

**Table 3:** Type of Considered Databases

Name of DB	Training set size [10^3^]	Considered quantity of images per molecule	Resulting number of data points in training set [10^3^]
QM9	120	1, 2, 4	120, 240, 480
QM9_2	60	1, 2, 4, 8	60, 120, 240, 480
QM9_3	40	1, 2, 4, 8, 12	40, 80, 160, 320, 480
QM9_4	30	1, 2, 4, 8, 16	30, 60, 120, 240, 480
QM9_6	20	1, 2, 4, 8, 16	20, 40, 80, 160, 320

To generate suitable data, we employed a Monte Carlo hyperparameter selection approach. Using a set of models trained on randomly selected parameter combinations, we performed a statistical analysis to derive meaningful observations and guide our conclusions.

### 4.1 Training Process

Our workflow was structured into distinct iterations, each involving a combination of hardcoded parameters and parameters selected randomly from specified ranges. Detailed information on this process is provided in Table S20.

### 4.2 Statistical Analysis

To determine whether differences within the group of searching parameters are statistically significant, we employed Analysis of Variance (ANOVA).^45^ Prior to conducting ANOVA, we verified the assumptions of normality and homogeneity of variances using the Shapiro-Wilk^46^ test and Levene test^47^ respectively. In cases where these conditions were not met, we used the Kruskal–Wallis test^48^ as the appropriate nonparametric alternative. If statistically significant differences were found (*p* < 0.05), we proceeded with posthoc analysis using Tukey’s HSD (Honestly Significant Difference) test or Dunn’s test.^49,50^ For continuous data, we look at the relation between the factor and accuracy visually, and based on that, we try to fit the corresponding model.

### 4.3 Software

All of this research was conducted using the code written in Python language. For building the training loop we used PyTorch^51^ and the fastai^52^ library. All statistics operations were performed using the statsmodel library.^53^ Plots have been generated using the matplotlib and seaborn Python libraries.^54,55^

## 5 Results and Discussion

The discussion is divided into two stages. In the first stage, we aim to test a wide range of model hyperparameter combinations to identify potential patterns and consequently constrain the search space. Subsequently, in the second stage, we conduct an in-depth analysis of the models/hyperparameter combinations that are expected to yield the highest accuracy.

Each model has been labeled as M* (e.g., M1, M74, M121). More details about training details have been provided in Table S21 and S22.

### 5.1 First Stage

#### 5.1.1 Impact of the Type of Database

(Figure 6, pink): Kruskall–Wallis test showed that there were no significant differences between analyzed databases. Therefore in the second stage, we analyze all of these types of databases. Outliers: M71 (374 meV, shuffling: none, training-set size: 160k) was the only A-S2CNN combination for QM9_3 cases, M98 (405 meV, shuffling: groups, training set size: 160k)—the only VGG19_bn architecture for QM9_3.

**Figure 6:**
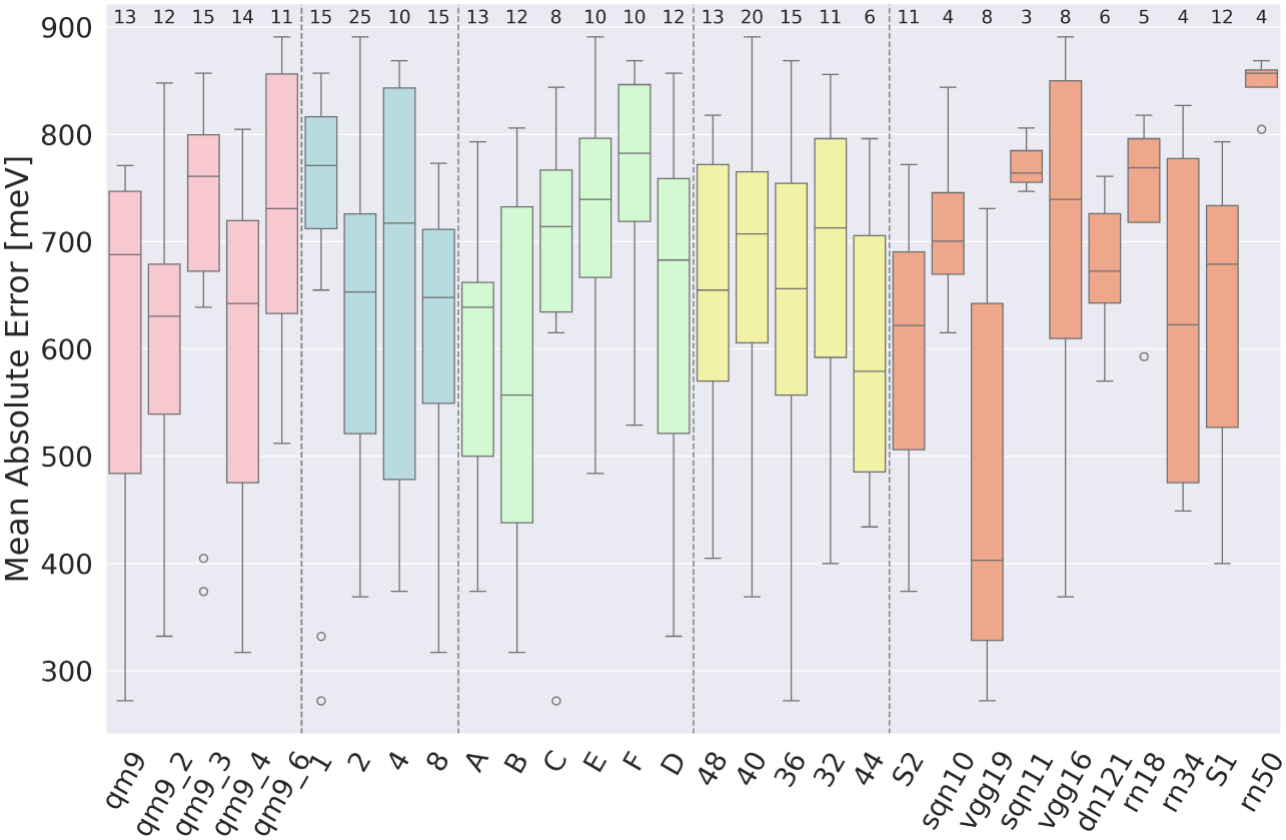
Influence of various parameters on the accuracy of the model. Different parameter categories are distinguished by color and separated by dashed lines. The parameters are listed from left to right: impact of the type of database (pink). Impact of the number of images generated per single molecule (blue). Impact of type of image generation algorithm (green). Impact of the final image size (yellow). Impact of the neural architecture (orange). For designations, see Table S21 and Table S22.

#### 5.1.2 Impact of the Number of Images Generated per Single Molecule

(Figure 6, blue): There were no significant differences between groups. Outliers: M44 (272 meV, shuffle: none, training set size: 120k), M105 (332 meV, shuffle: none, training set size: 60k, architecture: VGG19_bn), in this outliers there is only usage of VGG19_bn architecture for 1 image generated per single molecule.

#### 5.1.3 Number of Images in Training Set

(Figure 7, green) The differences between groups were statistically significant (ANOVA: 0.0036). Here, significant differences between groups: 40–160, 40–240, and 40–120 were observed. This prompted us to further investigate these parameters, as there is a noticeable difference between the least expansive model (in the context of images within the training set) and the three most frequent models, excluding the largest one. Nevertheless, we decided to test the largest model as well. Additionally, we incorporated the counts of 20 000 and 480 000 in our analysis. Outlier: M81 (512 meV) was the only one generated with the A method and the only S2CNN model in this category.

**Figure 7:**
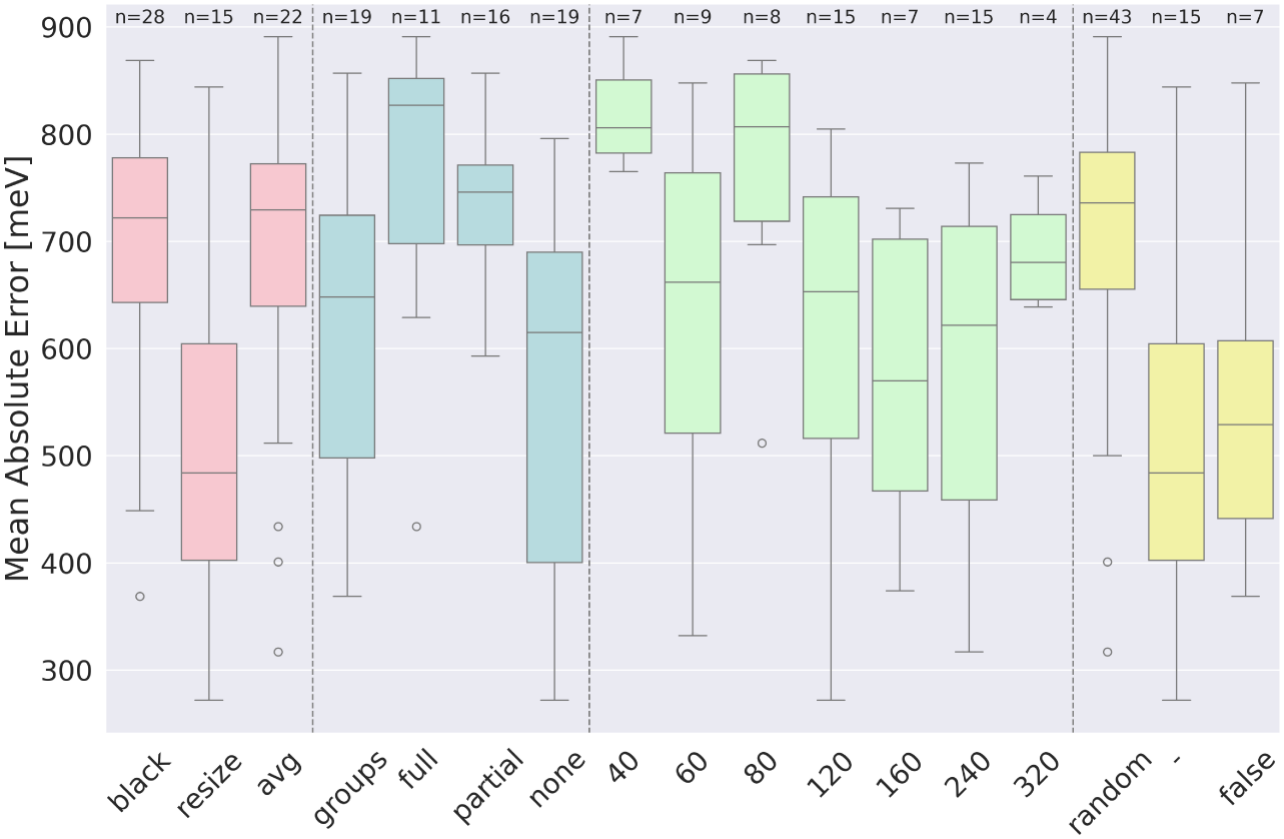
Influence of various parameters in the initial stage on the accuracy of the model (continuation). Different parameter categories are distinguished by color and separated by dashed lines. The consecutive parameters, listed from left to right: method used to adjust the size of images (pink). Shuffle types (blue). Number of images in the training set (green). Type of method used to randomize the offset of images (yellow). For designations, see Table S21 and Table S22.

#### 5.1.4 Impact of the Type of Image Generation Algorithm

(Figure 6, green): Although the ANOVA test revealed a significant difference, no pair of groups showed a statistically significant difference. For subsequent analysis, we selected the two algorithms with the lowest mean absolute error values (highest accuracy): Group A (mean = 598) and Group B (mean = 574), as these represent the most promising options based on their performance. Outliers: M44 (272 meV, shuffle: none, training set size: 120k) one generated making use of VGG19_bn architecture for 1 image generated per single molecule.

#### 5.1.5 Impact of the Size of the Final Image

(Figure 6, yellow): There were no statistically significant differences between groups. We conclude that the chemical information delivered through the image is nondependent of the size of the final image so this is the expected result.

#### 5.1.6 Impact of Neural Architecture

(Figure 6, orange): A significant difference was observed between VGG19_bn and both ResNet50 and ResNet18, supporting the choice of VGG19_bn as the primary neural architecture for further analysis. Additionally, VGG19_bn outperformed all other tested models in both mean and median values. No statistically significant differences were found among the remaining architectures. Despite this, S2CNN was selected as the second-best architecture for further analysis. It achieved a mean value of 595 meV and a median of 622 meV, ranking just below VGG19_bn. This makes S2CNN a strong candidate, as it effectively balances simplicity and performance. Outliers: M63 (593 meV, training set size: 240k, random_or = false) was the only one with the ″false″ setting with ResNet18, M101 (805 meV, training set size: 120k) used the largest set size for ResNet50.

#### 5.1.7 Method Used to Adjust the Size of Images

(Figure 7, pink): The differences between the groups were statistically significant (Kruskal–Wallis test: *p* = 0.0018). A posthoc analysis revealed a significant difference between the *resize-avg* and *resize-black* groups, with models based on the *resize* technique demonstrating better performance. Based on these results, we decided to proceed with this method in the second stage of the research. Outliers in ″avg″, ordered from the best one: M55 (no shuffling and 240k training set, random_or = random, 317 meV), M39 (no shuffling and 240k training set, random_or = random, 401 meV), and M74 (full shuffling, 240k training set, random_or = false, 434 meV). Outlier in ″black″ is M57 (369 meV, 240k training set, random_or = false, groups shuffling).

#### 5.1.8 Method Used to Randomize the Offset of the Image

(Figure 7, yellow): Results obtained for random offset differ significantly from both ″false″ (top-left corner), and ″resize″ filling techniques, where this functionality has no contribution. However, due to the nonchemical nature of this image property and the selection of the *resize* technique, we decided to proceed exclusively with images where the position adjustment was not applicable. Outliers: M39 (no shuffling and 240k training set, random_or = random, 401 meV) and M55 (no shuffling and 240k training set, random_or = random, 317 meV). Both were also the outliers in adjusting the size of images.

#### 5.1.9 Shuffle Types

(Figure 7, blue): For shuffling methods other than no-shuffling (see Section 3.4.2), it is possible to generate multiple unique representations for a single molecule. This represents a significant advantage of RGBChem, as most methods for predicting quantum properties often do not allow for data set augmentation. This capability is particularly beneficial for tasks where the available data set size is limited, providing an opportunity to enhance model performance through data diversity. Statistically significant differences were observed (Kruskall-Wallis: p-value = 0.0008) between the full–groups, full–none, and none–partial shuffling types. As expected, the most pronounced differences were found between the ″full″ and ″none″ shuffling techniques (on average: models with no-shuffling technique reach 205 meV better results in terms of mean absolute error). This can be attributed to the fact that the images generated with full shuffling were more similar to random noise rather than any well-structured data, making it difficult for the model to capture meaningful patterns. Similarly, the differences between partial shuffling and no-shuffling are both statistically significant, with a difference of 177 meV in favor of the models with no shuffling technique. Importantly, from the perspective of optimal model design, the difference between group shuffling and no-shuffling is negligible, with the p-value = 1 and average difference = 61.3 meV. This leads to the conclusion that the ″groups″ shuffling technique is the most effective method, as it simultaneously ensures permutation invariance, delivers high prediction quality, and allows for the generation of multiple images from a single molecule. Additionally, we decided that no-shuffling technique would not be further analyzed, even though it achieved the best average results. Models trained on arbitrarily ordered atoms tend to perform better, likely due to the artificial consistency introduced by the fixed order—which is expected in the case of CNN architectures. As a result, the model may attempt to learn chemically irrelevant patterns based on the arbitrary atom order used for constructing the image. Outlier: M74 (434 meV, full shuffle, 240k, random_or = false).

#### 5.1.10 Learning Rate, Momentum, Patience, and Batch Size

(Figure 8) influence on the results is shown in Figure 8. There is no clear pattern that could be reproduced, for instance, for linear regression for all of these plots, the p-value is above 0.05 (0.25, 0.18, 0.37, and 0.81, respectively) and *R* ^2^ is far below 0.1 (0.005, 0.015, 0.003, and 0.015, respectively). As none of these parameters is advantageous, we decided to use similar ranges of these parameters in the second stage of the analysis.

**Figure 8:**
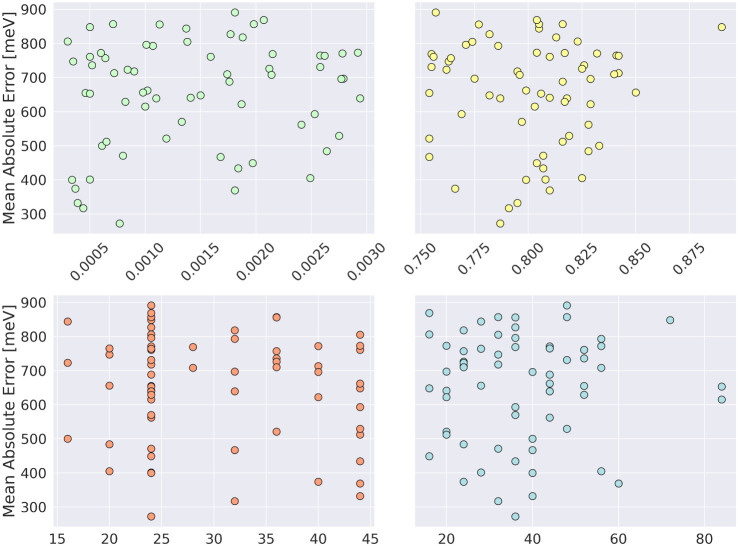
Impact of the learning rate (green), momentum (yellow), patience (orange), and batch size (blue) on the accuracy of the model for the initial stage of the study.

#### 5.1.11 Outliers

(Figures 7 and 6) Unexpectedly, well-performing models identified at this stage were characterized by the ″none″ shuffling techniques and/or the ″random_or″ parameter being set to false. Notably, the M81 model does not possess these attributes, and its apparent outlier status in terms of accuracy seems to arise more from the suboptimal performance of other models within the group rather than from the exceptional accuracy of the M81 model itself. A similar situation occurs for M101.

### 5.2 Second Stage

In the second stage of the research, we refine the parameter ranges based on the results obtained in the first stage, allowing for a more focused and extensive exploration within narrower intervals in order to find their optimal combination. Some sets of parameters are restricted—for example, the selection of neural architectures is reduced from ten to the two best-performing models identified in the first stage; others are strictly fixed to a single optimal value, eliminating further analysis in the second stage. Consequently, aspects such as the method for adjusting image size, the approach for randomizing image offset, and shuffling techniques are no longer considered, as they are unequivocally the ″resize″ and the group shuffle technique yielded the best results while preserving permutation invariance. The specific parameter ranges established in the first stage are detailed in Section 5.1.

#### 5.2.1 Impact of the Type of Database

(Figure 9, pink): the differences are significant for QM9–QM9_6, QM9_2–QM9_6, and QM9_3 and QM9_6 databases, in favor of the larger one. It appears that the QM9_6 database is not large enough to reach an accuracy similar to that of more complete databases. However, models trained on QM9_4, QM9_3, and QM9_2 databases, statistically do not differ from full QM9 database.

**Figure 9:**
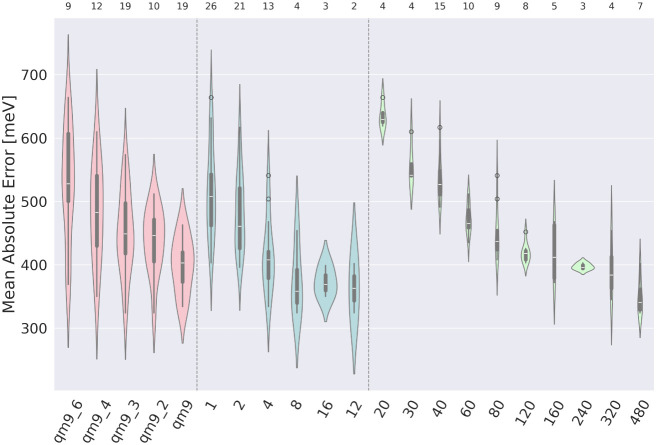
Influence of key parameters in the second stage of the research on the accuracy of the model. Different parameter categories are distinguished by color and separated by dashed lines. The consecutive parameters, listed from left to right, are as follows: impact of database (pink). Impact of number of images generated per molecule (blue). Impact of the number of images in the training set (green). For designations, see Table S21 and Table S22.

#### 5.2.2 Impact of the Number of Images Generated per Molecule

(Figure 9, blue): there are significant differences between many different combinations (e.g., 1–12, 1–16, 1–8, 2–8, 2–16). The overall tendency is that models where the number of molecules generated per image is 1 or 2 are worse than models where the number of molecules generated per image is 16, 12, 8, or even 4 (more details in Table S14). These results clearly show that these features are advantageous and can be used in other research suffering from limited data points. Outliers for 4 images per molecule: M135 (504 meV), M150 (541 meV) both are models trained on QM9_6 database and have relatively low learning rate (0.00031 and 0.00041, respectively); for 8 images per molecule: M126 (454 meV, only VGG19_bn architecture in the group, the smallest batch size in the group (28), and the shortest time of training in the group (78 epochs).

#### 5.2.3 Impact of the Type of Image Generation Algorithm and the Size of the Final Image

(Figure S1, green and yellow, respectively): No significant differences were observed between the groups. A similar conclusion applies to the characteristics of both A and B types of generated images, as seen in the analysis of neural architecture types. It is worth noting that for models trained using procedure A, significantly lower precision was achieved, producing both inferior and superior outcomes relative to models utilizing procedure B.

Outliers: for 40 × 40 pixels: M163 (632 meV, QM9_6 and only 20k training set size), for 48 × 48 pixels: M174 (627 meV, again QM9_6 and only 20k training set size).

#### 5.2.4 Number of Images in Training Set

(Figure 9, green): Kruskall-Wallis test showed significant differences between many groups (e.g. 20–120, 20–320, 20–480, 30–480, 40–120, 40–320, 40–480, and 60–480, more details in Table S14). Based on this and visual validation, we can conclude that increasing the number of data points by rearranging the atoms in the image representation can lead to better results in terms of the accuracy of the model. It is assumed that the improvement in prediction accuracy resulting from an increasing number of images generated per molecule is not unlimited, and at some point, it merely leads to prolonged training time without substantial gains in model performance. Our results confirm the overall trend of increasing accuracy with a higher number of images per molecule; however, the magnitude of this improvement diminishes progressively. Determining the number of images beyond which further increases do not contribute to prediction quality is not possible based on the data currently available and will be the subject of future investigations.

Analyzing the interaction effects between paired factors—specifically, the number of images in the training set and the type of database, as well as the number of images generated per single molecule and the type of database-does not yield conclusions that differ from those obtained when examining these parameters individually. Therefore, we conclude that the positive impact of increasing the number of data points is independent of the type of database.

Outliers: for 20k test size: M163 (664 meV, the highest patience in the group: 44, the lowest learning rate in the group: 0.00088; for 30k: M149 (610 meV, the lowest learning rate in the group: 0.00035; for 40k: M171 (617 meV; again the lowest learning rate in the group: 0.00038; for 80k: M135 (504 meV, lowest learning rate value: 0.00031), M150 (541 meV, second lowest learning rate value (0.00041) both of these model have been trained based on the QM9_6 database; for 120k the probable reason could not be determined.

#### 5.2.5 Impact of Neural Architecture

(Figure S1, orange): The performance of VGG19_bn and S2CNN in terms of accuracy is comparable. While S2CNN exhibits a broader interquartile range (IQR) relative to VGG19_bn, indicating greater variability in model performance, the upper-bound models within the VGG19_bn architecture achieve higher accuracy compared to those of S2CNN. Despite these differences, the median accuracy values of the two architectures remain statistically similar, suggesting no significant disparity in their overall performance.

An interesting observation is that VGG19_bn requires a longer training duration to achieve comparable accuracy levels. In the second stage of the research, the average number of epochs per model was 144, with S2CNN models averaging 112 epochs and VGG19_bn models requiring 179 epochs. This difference becomes more pronounced when analyzing the training duration for higher-performing models. For models achieving an average accuracy below 400 meV, S2CNN required on average 165 epochs, whereas VGG19_bn needed 372 epochs. Furthermore, the average number of data points processed per model was 155 000 for S2CNN and 141 000 for VGG19_bn. These findings underscore that the more complex VGG19_bn architecture requires significantly more time to learn meaningful patterns and converge to a minimum. This observation suggests that, for this type of task, simpler architectures, such as S2CNN, are more advantageous as they provide noticeably faster training times while maintaining comparable accuracy to more advanced models.

#### 5.2.6 Learning Rate, Momentum, Patience, Batch Size

(Figure S6): Similarly to the first stage, no clear patterns were observed, which could indicate the optimal value.

#### 5.2.7 Efficacy of Expanding the Training Set

For all shuffling techniques except ″none″ (Figure 3), it is possible to generate more than one unique image per molecule. However, we also trained models using the ″none″ shuffling technique with multiple images per molecule. To determine whether the positive impact of increasing the number of images per molecule on model accuracy is specific to the ″groups″, ″partial″, and ″full″ shuffling techniques, we compared the results between models trained with the ″none″ and ″groups″ shuffling approaches. The outcomes for the no-shuffling technique are presented in Figure S4, while the results for the groups shuffling technique are shown in Figure 9 (green). This comparison demonstrates that the ″groups″ shuffling technique benefits from increasing the number of images per molecule, whereas the ″none″ does not exhibit a similar trend.

## 6 Conclusions

In this work, we introduce RGBChem, a novel approach for converting chemical compounds into image representations, subsequently used to train convolutional neural networks (CNNs) for predicting the HOMO–LUMO gap.

Through testing of over a hundred training configurations, it was determined that the ″groups″ shuffling technique, combined with the vgg19_bn and S2CNN neural network architectures, yielded the highest model accuracy. In contrast, many tested parameters, such as the type of image generation algorithm, batch size, learning rate, and momentum, had minimal impact on the model’s performance.

To address the problem of artificial order present in .xyz files used for image generation, three approaches with varying levels of entropy were evaluated. These included the ″groups″ shuffling technique, which involves relatively few atom order permutations, and the full shuffling technique, characterized by a high degree of randomness resembling a disordered visualization system. The results indicate that the ″groups″ shuffling technique demonstrates greater potential compared to both partial and full shuffling methods, while also preserving the permutational invariance of the generated representations.

By leveraging shuffling techniques, multiple unique data points were generated from a single molecule, effectively expanding the size of the training data set. This approach was shown to lead to statistically significant improvements in model accuracy, positioning RGBChem as a valuable tool for applying machine learning methods in scenarios where data availability is a limiting factor.

## 7 Future Work

Future work may explore the effectiveness of the proposed workflow under conditions of significantly reduced data availability, ranging from several dozen to a few hundred molecules. Additionally, scenarios involving an increased number of images generated per molecule (e.g., 32, 64, or more images per molecule) could be investigated. Further research might also examine the potential applicability of RGBChem to chemical data sets beyond simple organic compounds from the QM9 database, such as data sets of organometallic compounds.

Investigating other types of chemical information that can be utilized for image generation (e.g., ACSF^56^) and evaluating how the integration of CNNs with attention-based neural network architectures impacts the accuracy of the approach also may be a valuable area of further research.

## Supplementary Material



## Data Availability

Detailed results of the statistical analysis are collected in the Supporting Information. The source code is available as a Code Ocean capsule at 10.24433/CO.3284039.v1.

## Notes

Statistical test results, post-hoc results, tested parameters, types of trained models for bandwidth prediction, violin plots (PDF)
