# Supplementary material for: RGBChem: Image-Like Representation of Chemical Compounds for Property Prediction
Source: J Chem Theory Comput. 2025 May 12;21(10):5322–33. doi: 10.1021/acs.jctc.5c00291 (PMC12120917; doi:10.1021/acs.jctc.5c00291)
Supplement: Supplementary file 1 [file ct5c00291_si_001.pdf]

RGBChem: Image-like Representation of Chemical Compounds for Properties Prediction – Supplementary Information

Rafał Stottko[1,*], Radosław Michalski[2], Bartłomiej M. Szyja[1,*]

[1] Institute of Advanced Materials, Wrocław University of Science and Technology, Gdańska 7/9, 50-344 Wrocław, Poland

[2] Department of Artificial Intelligence, Wrocław University of Science and Technology, Wyb. Wyspiańskiego 27, 50-370 Wrocław, Poland

[*] E-mail: rafal.stottko@pwr.edu.pl, b.m.szyja@pwr.edu.pl

List of Tables

S1 Statistical test results (p-value) for the initial stage of the study comparing methods for image size adjustment (Figure 7, pink) S10
S2 Statistical test results (p-values) for the initial stage of the study comparing shuffling techniques (Figure 7, blue) S11
S3 Statistical test results (p-values) for the initial stage of the study comparing method used to randomize orientation of images (Figure 7, yellow) S12
S4 Statistical test results (p-values) for the initial stage of the study comparing type of databases (Figure 6, pink) S13
S5 Statistical test results (p-values) for the initial stage of the study comparing number of images in training set (Figure 7, green) S14
S6 Statistical test results (p-values) for the initial stage of the study comparing number of images generated per single molecule (Figure 6, blue) S15
S7 Statistical test results (p-values) for the initial stage of the study comparing types of image generation algorithms (Figure 6, green) S16
S8 Statistical test results (p-values) for the initial stage of the study comparing final size of images (Figure 6, yellow) S17
S9 Statistical test results (normality, homogeneity of variance, and ANOVA) for the initial stage of the study comparing neural network architectures, p-values (Figure 6, orange) S18
S10 Post–hoc results for pairwise architecture comparison (initial stage of study, p-values), (Figure 6, orange) S19
S11 Statistical test results (normality, homogeneity of variance, and ANOVA) for the second stage of the study comparing types of databases (Figure 9, pink) S20
S12 Post–hoc results for pairwise databases comparison (second stage of study, Figure 9, pink) S21
S13 Statistical test results (normality, homogeneity of variance, and ANOVA) for the second stage of the study comparing different numbers of images generated per single molecule (Figure 9, blue) S22
S14 Post–hoc results for pairwise different number of images generated per single molecule comparison (second stage of study, Figure 9, blue) S23

S15 Statistical test results (normality, homogeneity of variance, and Kruskal–Wallis) for the second stage of the study comparing two image generation algorithm (A and B, Figure 9, yellow) . . . S24
S16 Statistical test results (normality, homogeneity of variance, and Kruskal–Wallis) for the second stage of the study comparing S2CNN and VGG19_bn types of neural network architecture, (Figure 9, orange) . . . S25
S17 Statistical test results (normality, homogeneity of variance, and ANOVA) for the second stage of the study comparing different final images sizes, (Figure 9, yellow) . . . S26
S18 Statistical test results (normality, homogeneity of variance, and ANOVA) for the all models where none shufling techniques have been applied. . . . S27
S19 Statistical test results (normality, homogeneity of variance, and Kruskal–Wallis) for the second stage of the study comparing different numbers of images in training set, (Figure 9, pink) . . . S28
S20 The ranges of tested parameters included network architecture, batch size, learning rate, momentum, and patience for the machine learning–related parameters. For image generation, the parameters spanned database selection, the number of images generated per molecule, the size of the training set, the type of image generation algorithm, margin type, image size, margin orientation, and shuffle type. All models was trained to predict the HOMO-LUMO gap energy. . . . S29
S21 Type of all trained models for prediction of bandgap (for first stage of research) . . . S30
S22 Type of all trained models for prediction of bandgap (for second stage of research) . . . S32

List of Figures

S1 Influence of not significant parameters in the second stage of the research on the accuracy of the model. Different parameter categories are distinguished by color and separated by dashed lines. The consecutive parameters, listed from left to right, are as follows: type of image generation algorithm (green), impact of image size (yellow) and impact of type of neural network architecture (orange). For designations see Table S21 and Table S22 . . . S4
S2 Violin plot illustrating the impact of the number of images in the training set during the initial stage of the study for models in which the shuffling technique was not applied. . . . S5
S3 Violin plot illustrating the impact of the number of images in the training set during the initial stage of the study for models in which the shuffling technique was not applied. . . . S6

S4 Violin plot alternative to box plot (Fig. 7). Influence of various parameters in the initial stage on the accuracy of the model (continuation). Different parameter categories are distinguished by color and separated by dashed lines. The consecutive parameters, listed from left to right: Method used to adjust the size of images (pink). Shuffle types (blue). Number of images in the training set (green). Type of method used to randomize the offset of images (yellow). For designations see Table S21 and Table S22. . . . . S7
S5 Violin plot alternative to box plot (Fig. 6). Influence of various parameters on the accuracy of the model. Different parameter categories are distinguished by color and separated by dashed lines. The parameters are listed from left to right: Impact of the type of database (pink). Impact of the number of images generated per single molecule (blue). Impact of type of image generation algorithm (green). Impact of the final image size (yellow). Impact of neural architecture (orange). For designations see Table S21 and Table S22 . . . . S8
S6 Impact of the learning rate (green), momentum (yellow), patience (orange), batch size (blue) on the accuracy of the model. Similar to the previous iteration there is no clear pattern that we can reproduce (for linear regression for all of these plots p-value is above 0.05 (0.053, 0.50, 0.36, 0.53, respectively), and R^2 is far below 0.1 (0.04, 0.008, 0.002, 0.009, respectively) so we conclude that there is no obvious pattern which we can follow and any values from given ranges as similarly good. S9

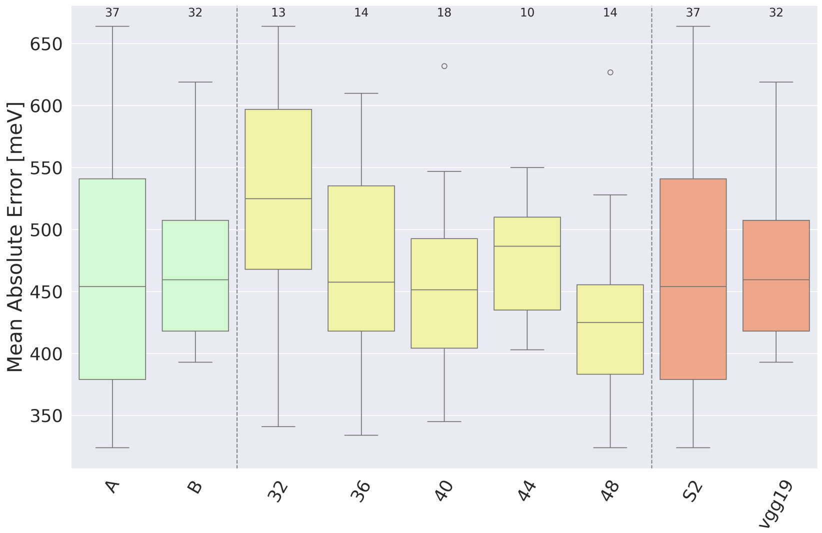

Figure S1: Influence of not significant parameters in the second stage of the research on the accuracy of the model. Different parameter categories are distinguished by color and separated by dashed lines. The consecutive parameters, listed from left to right, are as follows: type of image generation algorithm (green), impact of image size (yellow) and impact of type of neural network architecture (orange). For designations see Table S21 and Table S22

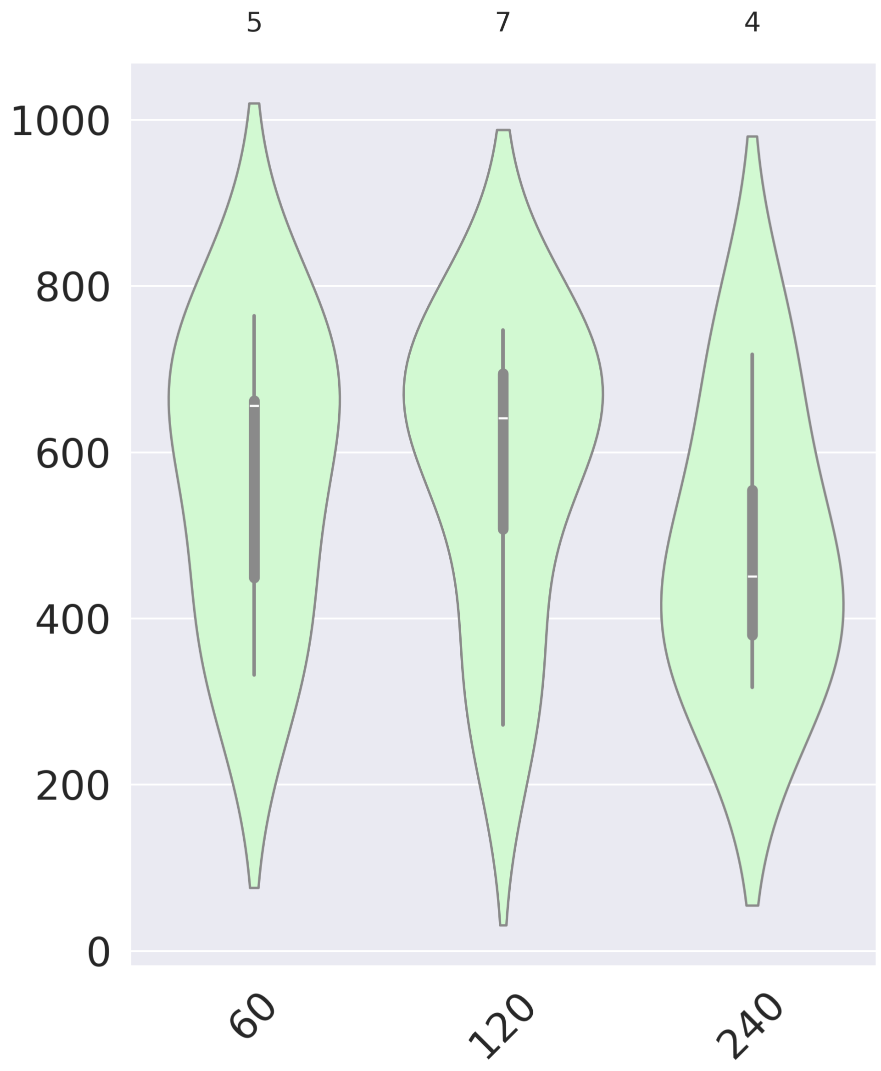

Figure S2: Violin plot illustrating the impact of the number of images in the training set during the initial stage of the study for models in which the shuffling technique was not applied.

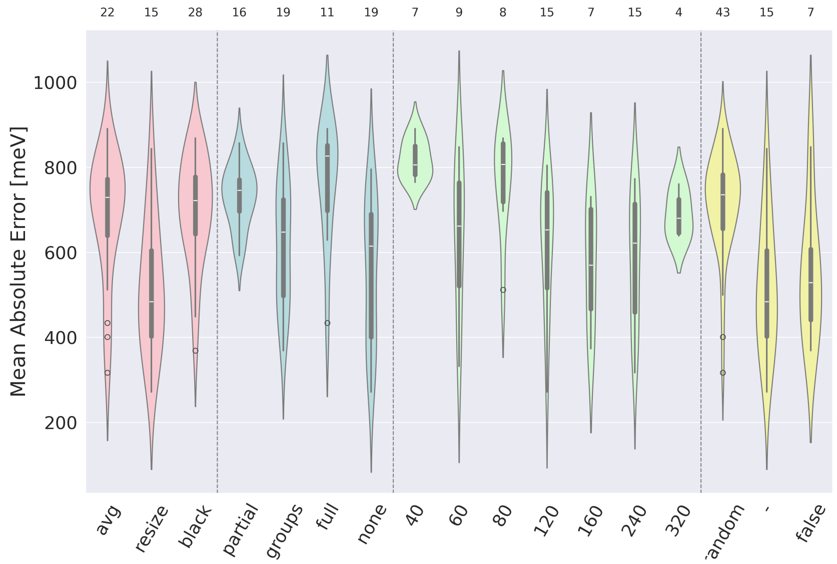

Figure S3: Violin plot illustrating the impact of the number of images in the training set during the initial stage of the study for models in which the shuffling technique was not applied.

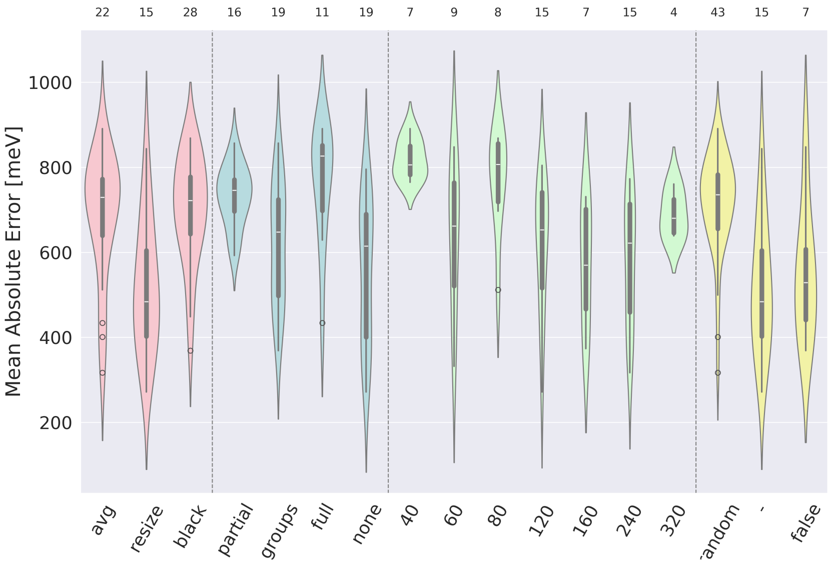

Figure S4: Violin plot alternative to box plot (Fig. 7). Influence of various parameters in the initial stage on the accuracy of the model (continuation). Different parameter categories are distinguished by color and separated by dashed lines. The consecutive parameters, listed from left to right: Method used to adjust the size of images (pink). Shuffle types (blue). Number of images in the training set (green). Type of method used to randomize the offset of images (yellow). For designations see Table S21 and Table S22.

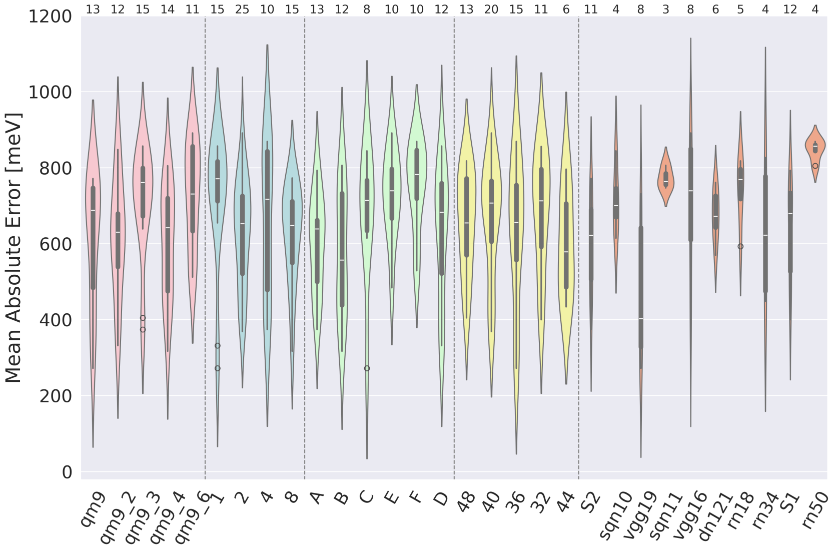

Figure S5: Violin plot alternative to box plot (Fig. 6). Influence of various parameters on the accuracy of the model. Different parameter categories are distinguished by color and separated by dashed lines. The parameters are listed from left to right: Impact of the type of database (pink). Impact of the number of images generated per single molecule (blue). Impact of type of image generation algorithm (green). Impact of the final image size (yellow). Impact of neural architecture (orange). For designations see Table S21 and Table S22

.

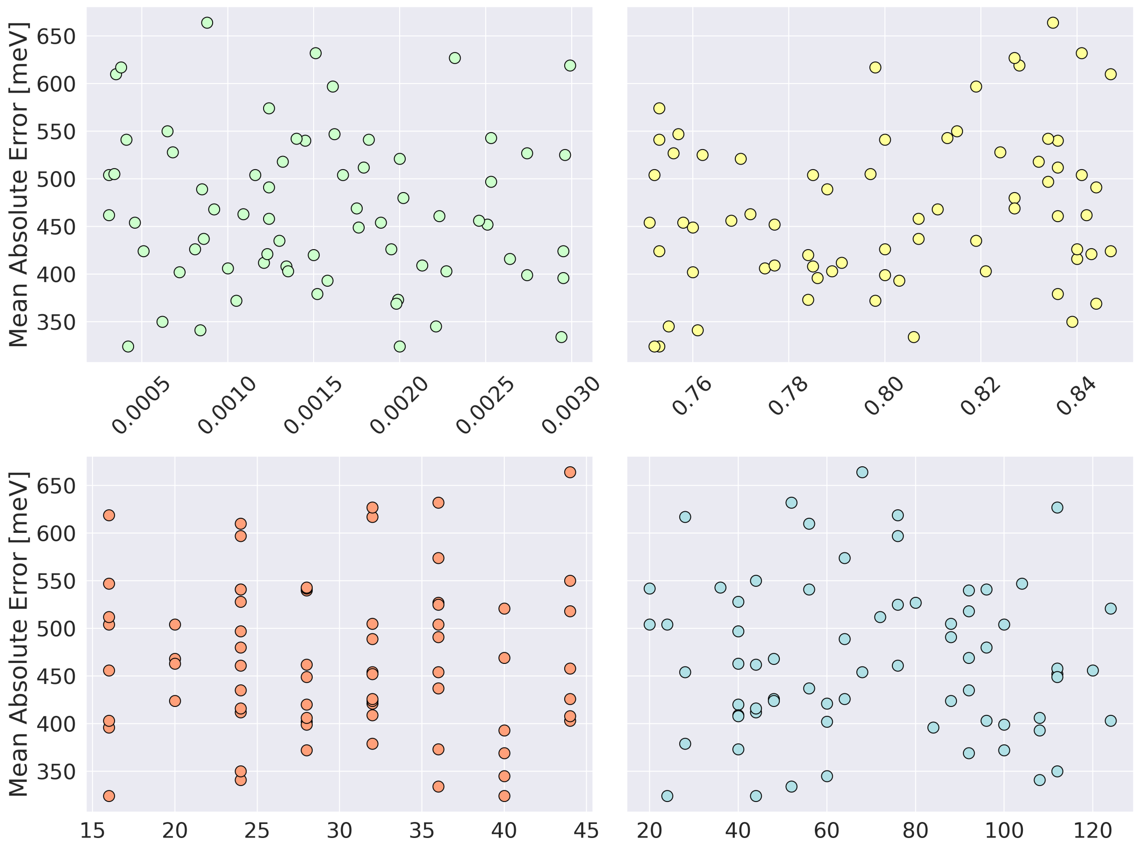

Figure S6: Impact of the learning rate (green), momentum (yellow), patience (orange), batch size (blue) on the accuracy of the model. Similar to the previous iteration there is no clear pattern that we can reproduce (for linear regression for all of these plots p-value is above 0.05 (0.053, 0.50, 0.36, 0.53, respectively), and R^2 is far below 0.1 (0.04, 0.008, 0.002, 0.009, respectively) so we conclude that there is no obvious pattern which we can follow and any values from given ranges as similarly good.

Table S1: Statistical test results (p-value) for the initial stage of the study comparing methods for image size adjustment (Figure 7, pink)

**Group**	**Shapiro–Wilk test**	**Post-hoc Dunn Test (p-values)**		
		avg	black	resize
avg	0.0110	1.0000	1.0000	0.0066
black	0.1069	1.0000	1.0000	0.0026
resize	0.9092	0.0066	0.0026	1.0000
**Levene test**	0.7616			
**Kruskall-Wallis test**	0.0017			

Table S2: Statistical test results (p-values) for the initial stage of the study comparing shuffling techniques (Figure 7, blue)

**Group**	**Shapiro–Wilk test**	**Post-hoc Dunn Test (p-values)**			
		full	groups	none	partial
full	0.0217	1.0000	0.0481	0.0031	1.0000
groups	0.5542	0.0481	1.0000	1.0000	0.2322
none	0.1347	0.0031	1.0000	1.0000	0.0172
partial	0.7942	1.0000	0.2322	0.0172	1.0000
**Levene test**	0.0210				
**Kruskall-Wallis test**	0.0008				

Table S3: Statistical test results (p-values) for the initial stage of the study comparing method used to randomize orientation of images (Figure 7, yellow)

**Group**	**Shapiro–Wilk test**	**Post-hoc Dunn Test (p-values)**		
		false	random	–
false	0.5068	1.0000	0.0273	1.0000
random	0.0008	0.0273	1.0000	0.0002
–	0.9092	1.0000	0.0002	1.0000
**Levene test**	0.3086			
**Kruskall Wallis test**	6×10^{-5}			

Table S4: Statistical test results (p-values) for the initial stage of the study comparing type of databases (Figure 6, pink)

Group	Shapiro–Wilk test
qm9	0.0231
qm9_2	0.6721
qm9_3	0.0060
qm9_4	0.4081
qm9_6	0.1466
**Levene test**	0.9114
**Kruskall Wallis test**	0.0743

Table S5: Statistical test results (p-values) for the initial stage of the study comparing number of images in training set (Figure 7, green)

**Group**	**Shapiro–Wilk test**	**Post-hoc Dunn Test (p-values)**			
		2nd group	Mean Diff.	p–adj	$p < 0.05$
40	0.6376	60	-175.84	0.1916	False
		80	-51.91	0.9916	False
		120	-202.28	0.0420	True
		160	-245.00	0.0309	True
		240	-237.02	0.0094	True
		320	-128.04	0.7763	False
60	0.5538	80	123.93	0.5525	False
		120	-26.44	0.9994	False
		160	-69.16	0.9586	False
		240	-61.18	0.9461	False
		320	47.80	0.9976	False
80	0.0628	120	-150.38	0.2066	False
		160	-193.09	0.1344	False
		240	-185.11	0.0593	False
		320	-76.12	0.9745	False
120	0.1942	160	-42.71	0.9943	False
		240	-34.73	0.9937	False
		320	74.25	0.9657	False
160	0.2801	240	7.98	1.0000	False
		320	116.96	0.8407	False
240	0.2389	320	108.98	0.8162	False
320	0.4406				
**Levene test**	0.2218				
**ANOVA test**	0.0036				

Table S6: Statistical test results (p-values) for the initial stage of the study comparing number of images generated per single molecule (Figure 6, blue)

**Group**	**Shapiro–Wilk test**
1	0.0003
2	0.4395
4	0.0579
8	0.0986
**Levene test**	0.2594
**Kruskal–Wallis test**	0.0732

Table S7: Statistical test results (p-values) for the initial stage of the study comparing types of image generation algorithms (Figure 6, green)

**Group**	**Shapiro–Wilk test**	**Post-hoc Dunn Test (p-values)**			
		2nd group	Mean Diff.	p–adj	p < 0.05
A	0.5499	B	-23.01	0.9989	False
		C	73.83	0.8823	False
		D	31.58	0.9950	False
		E	128.18	0.3398	False
		F	153.38	0.1643	False
B	0.2913	C	96.83	0.7201	False
		D	54.58	0.9476	False
		E	151.18	0.1915	False
		F	176.38	0.0823	False
C	0.0544	D	-42.25	0.9895	False
		E	54.35	0.9727	False
		F	79.55	0.8730	False
D	0.1082	E	96.60	0.6651	False
		F	121.80	0.4170	False
E	0.8542	F	25.20	0.9990	False
F	0.0896				
**Levene test**	0.5944				
**ANOVA test**	0.0471				

Table S8: Statistical test results (p-values) for the initial stage of the study comparing final size of images (Figure 6, yellow)

**Group**	**Shapiro–Wilk test**
32	0.2225
36	0.1051
40	0.0350
44	0.6327
48	0.3266
**Levene test**	0.9188
**Kruskall Wallis test**	0.8421

Table S9: Statistical test results (normality, homogeneity of variance, and ANOVA) for the initial stage of the study comparing neural network architectures, p-values (Figure 6, orange)

**Group**	**Shapiro–Wilk test**
S1	0.2424
S2	0.7644
dn121	0.8581
r18	0.3177
r34	0.2265
r50	0.0859
sqn10	0.7368
sqn11	0.5418
vgg16	0.2648
vgg19	0.1467
**Levene test**	0.0722
**ANOVA test**	0.0021

Table S10: Post–hoc results for pairwise architecture comparison (initial stage of study, p-values), (Figure 6, orange)

Post-hoc Tukey HSD Test (sorted results)

Group1	Group2	Mean Diff.	p-adj	p < 0.05
r50	vgg19	-378.25	0.0013	True
r18	vgg19	-270.05	0.0321	True
vgg16	vgg19	-227.50	0.0463	True
sqn11	vgg19	-303.58	0.0524	False
S2	r50	252.18	0.0711	False
sqn10	vgg19	-246.25	0.1192	False
r34	r50	216.75	0.4426	False
S1	r50	207.16	0.2318	False
dn121	vgg19	-206.75	0.1624	False
S2	sqn11	177.51	0.6077	False
dn121	r50	171.50	0.6411	False
S1	vgg19	-171.08	0.1836	False
r34	vgg19	-161.50	0.6505	False
r50	vgg16	-150.75	0.7324	False
S2	r18	143.98	0.6349	False
r34	sqn11	142.08	0.9340	False
S1	sqn11	132.50	0.8861	False
r50	sqn10	-132.00	0.9326	False
S2	vgg19	-126.07	0.6137	False
S2	sqn10	120.18	0.8845	False
r18	r34	-108.55	0.9720	False
r18	r50	108.20	0.9726	False
S2	vgg16	101.43	0.8443	False
S1	r18	98.97	0.9342	False
dn121	sqn11	96.83	0.9910	False
r34	sqn10	84.75	0.9966	False
S2	dn121	80.68	0.9750	False
sqn11	vgg16	-76.08	0.9979	False
S1	sqn10	75.17	0.9937	False
r50	sqn11	-74.67	0.9993	False
r34	vgg16	66.00	0.9985	False
dn121	r18	63.30	0.9988	False
sqn10	snq11	57.33	0.9999	False
S1	vgg16	56.42	0.9957	False
dn121	r34	-45.25	1.0000	False
S1	S2	-45.01	0.9985	False
r18	vgg16	-42.55	0.9999	False
dn121	sqn10	39.50	1.0000	False
S1	dn121	35.67	0.9999	False
S2	r34	35.43	1.0000	False

Table S11: Statistical test results (normality, homogeneity of variance, and ANOVA) for the second stage of the study comparing types of databases (Figure 9, pink)

**Group**	**Shapiro–Wilk test**
qm9	0.7191
qm9_2	0.7014
qm9_3	0.9657
qm9_4	0.8827
qm9_6	0.1693
**Levene test**	0.1987
**ANOVA test**	0.0002

Table S12: Post–hoc results for pairwise databases comparison (second stage of study, Figure 9, pink)

**Group 1**	**Group 2**	**Mean Diff.**	**p-adj**	**p < 0.05**
qm9	qm9_6	127.2749	0.0004	True
qm9_2	qm9_6	88.9693	0.0099	True
qm9_3	qm9_6	76.4211	0.0126	True
qm9	qm9_4	83.7222	0.0888	False
qm9	qm9_3	50.8538	0.3993	False
qm9_2	qm9_4	45.4167	0.5697	False
qm9_4	qm9_6	43.5526	0.5213	False
qm9	qm9_2	38.3056	0.7375	False
qm9_3	qm9_4	32.8684	0.7596	False
qm9_2	qm9_3	12.5482	0.9890	False

Table S13: Statistical test results (normality, homogeneity of variance, and ANOVA) for the second stage of the study comparing different numbers of images generated per single molecule (Figure 9, blue)

**Group**	**Shapiro–Wilk test**
1	0.3253
2	0.2065
4	0.2491
8	0.4490
12	1.0000
16	0.7538
**Levene test**	0.5061
**ANOVA test**	2.14×10^{-6}

Table S14: Post–hoc results for pairwise different number of images generated per single molecule comparison (second stage of study, Figure 9, blue)

**Group 1**	**Group 2**	**Mean Diff.**	**p-adj**	**p < 0.05**
1	12	-152.31	0.0247	True
1	16	-142.64	0.0076	True
1	8	-141.56	0.0018	True
2	8	-109.25	0.0335	True
1	4	-101.00	0.0003	True
2	4	-68.69	0.0421	True
2	12	-120.00	0.1384	False
2	16	-110.33	0.0776	False
4	12	-51.31	0.9013	False
4	16	-41.64	0.9149	False
4	8	-40.56	0.8814	False
1	2	-32.31	0.5365	False
8	12	-10.75	1.0000	False
8	16	-1.08	1.0000	False
12	16	9.67	1.0000	False

Table S15: Statistical test results (normality, homogeneity of variance, and Kruskal–Wallis) for the second stage of the study comparing two image generation algorithm (A and B, Figure 9, yellow)

**Group**	**Shapiro–Wilk test**
A	0.1224
B	0.0509
**Levene test**	0.0012
**Kruskal–Wallis test**	0.8379

Table S16: Statistical test results (normality, homogeneity of variance, and Kruskal–Wallis) for the second stage of the study comparing S2CNN and VGG19_bn types of neural network architecture, (Figure 9, orange)

**Group**	**Shapiro–Wilk test**
S2CNN	0.1224
vgg19	0.0509
**Levene test**	0.0012
**Kruskal–Wallis test**	0.8379

Table S17: Statistical test results (normality, homogeneity of variance, and ANOVA) for the second stage of the study comparing different final images sizes, (Figure 9, yellow)

**Group**	**Shapiro–Wilk test**
32	0.5399
36	0.9825
40	0.4241
44	0.5877
48	0.3362
**Levene test**	0.4858
**ANOVA test**	0.1054

—

Table S18: Statistical test results (normality, homogeneity of variance, and ANOVA) for the all models where none shufling techniques have been applied.

Group	Shapiro–Wilk test
60	0.5216
120	0.1408
240	0.7160
**Levene test**	0.9949
**ANOVA test**	0.6641

Table S19: Statistical test results (normality, homogeneity of variance, and Kruskal–Wallis) for the second stage of the study comparing different numbers of images in training set, (Figure 9, pink)

**Group**	**Shapiro–Wilk test**	**Post-hoc Tukey HSD Test (p-values)**									
		20	30	40	60	80	120	160	240	320	480
20	0.2703	1.000	1.000	1.000	0.735	0.143	0.009	0.042	0.016	0.008	3.8e-05
30	0.0028	1.000	1.000	1.000	1.000	1.000	0.133	0.386	0.133	0.087	1.2e-03
40	0.1568	1.000	1.000	1.000	1.000	0.420	0.012	0.127	0.054	0.021	6.0e-06
60	0.6780	0.735	1.000	1.000	1.000	1.000	1.000	1.000	1.000	1.000	0.032
80	0.0568	0.143	1.000	0.420	1.000	1.000	1.000	1.000	1.000	1.000	0.409
120	0.1680	0.009	0.133	0.012	1.000	1.000	1.000	1.000	1.000	1.000	1.000
160	0.2380	0.042	0.386	0.127	1.000	1.000	1.000	1.000	1.000	1.000	1.000
240	0.5665	0.016	0.133	0.054	1.000	1.000	1.000	1.000	1.000	1.000	1.000
320	0.7967	0.008	0.087	0.021	1.000	1.000	1.000	1.000	1.000	1.000	1.000
480	0.2147	3.8e-05	1.2e-03	6.0e-06	0.032	0.409	1.000	1.000	1.000	1.000	1.000
**Levene test**	0.4209										
**Kruskal–Wallis test**	3.15×10^{-9}										

Table S20: The ranges of tested parameters included network architecture, batch size, learning rate, momentum, and patience for the machine learning–related parameters. For image generation, the parameters spanned database selection, the number of images generated per molecule, the size of the training set, the type of image generation algorithm, margin type, image size, margin orientation, and shuffle type. All models was trained to predict the HOMO-LUMO gap energy.

Stage	1	2
No tested models	65	69
Database	qm9, qm9_2, qm9_3, qm9_4, qm9_6	qm9, qm9_2, qm9_3, qm9_4, qm9_6
Quantity of images per molecule	1,2,4,8	1,2,4,8,12,16
Quantity of training set [10^3]	40-320	20-480
Image type	A, B, C, D, E, F	A,B
Shuffle type	partial, full, none, groups	groups
Margin type	black, resize, avg	resize
Image size	32,36,40,44,48	32,36,40,44,48
Margin orientation	random, no, - (for resize cases)	-
Architecture	resnet18, 34, 50, squeezenet1_0, densenet121, squeezenet1_1, vgg16_bn, vgg19_bn, S1CNN, S2CNN	S2CNN, vgg19_bn
Batch size	16-84	16-128
Learning rate (LR)	3e-4 – 3e-3	3e-4 – 3e-3
Momentum	0.75–0.9	0.75–0.9
Patience	16-48	16-48
Accuracy of model (mae, meV)	272-891	324-664

Table S21: Type of all trained models for prediction of bandgap (for first stage of research)

ID	DB, Number of images per molecule, training set size[10^3], image generation type , shuffling type	Architecture, Epochs, Batch size	Margins (type, size and orientation)	LR, Momentum, Patience, Accuracy of model mae, meV
M1	qm9,2 , 240,B, partial	r34, 34, 52	black , 40, random	0.0005, 0.81, 24 ,761
M3	qm9_3,1 , 40,B, partial	sqn11, 93, 16	avg, 48, random	0.0003, 0.823, 24 ,806
M4	qm9_2,2 , 120,C, none	sqn10, 146, 84	black , 48, random	0.001, 0.803, 24 ,615
M5	qm9_6,6 , 120,A, partial	vgg19, 39, 84	black , 40, random	0.001, 0.807, 24 ,757
M7	qm9_2,1 , 60,E, full	vgg16, 39, 72	black , 32, false	0.0005, 0.89, 24 ,848
M8	qm9_2,2 , 120,B, groups	vgg16, 185, 32	resize, 44,-	0.0008, 0.807, 24 ,471
M9	qm9_2,2 , 120,E, none	vgg19, 44, 84	black , 48, random	0.0005, 0.806, 24 ,653
M38	qm9_2,6 , 360,C, full	r34, 32, 44	black , 32, random	0.0005, 0.854, 24 ,804
M39	qm9,2 , 240,B, none	vgg19, 117, 28	avg, 40, random	0.0005, 0.808, 24 ,401
M40	qm9_4,2 , 60,B, none	sqn11, 108, 28	avg, 40, random	0.00262, 0.842, 24 ,764
M41	qm9,2 , 240,E, none	r18, 45, 32	black , 40, random	0.0009, 0.795, 24 ,718
M42	qm9_6,8 , 160,C, partial	vgg19, 33, 48	black , 44, random	0.00258, 0.755, 24 ,731
M44	qm9,1 , 120,C, none	vgg19, 193, 36	resize, 36,-	0.00077, 0.787, 24 ,272
M45	qm9_2,1 , 60,C, full	r34, 39, 36	avg, 32, random	0.00177, 0.782, 24 ,827
M47	qm9_6,2 , 40,E, full	vgg16, 36, 48	avg, 40, random	0.00181, 0.757, 24 ,891
M48	qm9_4,2 , 60,B, none	r34, 120, 16	black , 32, false	0.00197, 0.804, 24 ,449
M49	qm9_6,8 , 160,F, groups	densenet121, 208, 36	resize, 48,-	0.00133, 0.797, 24 ,570
M50	qm9_6,4 , 80,D, partial	r50, 38, 48	black , 40, random	0.00198, 0.816, 24 ,857
M51	qm9,1 , 120,E, partial	vgg16, 35, 44	avg, 40, random	0.00278, 0.832, 24 ,771
M52	qm9_6,4 , 80,F, full	r50, 45, 16	black , 36, random	0.00207, 0.804, 24 ,869
M53	qm9_6,4 , 80,F, none	r18, 39, 36	black , 44, random	0.00101, 0.771, 24 ,796
M54	qm9_2,2 , 120,C, none	densenet121, 59, 20	avg, 40, random	0.00141, 0.81, 24 ,641
M55	qm9_4,8 , 240,B, none	vgg19, 515, 32	avg, 36, random	0.00044, 0.791, 32 ,317
M56	qm9_3,1 , 40,C, full	sqn10, 57, 28	resize, 36,-	0.00137, 0.805, 16 ,844
M57	qm9,2 , 240,D, groups	vgg16, 637, 60	black , 40, false	0.00181, 0.81, 44 ,369
M58	qm9_2,2 , 120,D, full	S2, 67, 24	avg, 40, random	0.00064, 0.764, 36 ,757
M59	qm9_3,1 , 40,F, groups	r50, 64, 32	black , 36, random	0.00071, 0.805, 36 ,857
M61	qm9,2 , 240,F, partial	r18, 38, 36	avg, 40, random	0.00215, 0.755, 28 ,769
M62	qm9_3,2 , 80,F, partial	r18, 50, 24	black , 48, random	0.00188, 0.813, 32 ,818
M63	qm9,2 , 240,B, partial	r18, 71, 36	black , 36, false	0.00253, 0.769, 44 ,593
M64	qm9_6,4 , 80,F, full	vgg16, 44, 36	black , 32, random	0.00113, 0.777, 36 ,856
M67	qm9,1 , 120,A, groups	S2, 64, 52	black , 48, random	0.00046, 0.754, 24 ,655
M68	qm9_3,1 , 40,A, groups	S2, 44, 56	avg, 48, random	0.00107, 0.786, 32 ,793
M69	qm9_4,8 , 240,A, full	S2, 59, 20	avg, 36, random	0.00292, 0.804, 44 ,773
M70	qm9_6,8 , 160,F, none	S2, 225, 48	black , 44, false	0.00275, 0.819, 44 ,529
M71	qm9_3,4 , 160,A, none	S2, 149, 24	resize, 40,-	0.00037, 0.766, 40 ,374
M72	qm9_3,8 , 320,A, full	S2, 54, 44	resize, 36,-	0.00294, 0.787, 24 ,639
M73	qm9_3,1 , 40,D, partial	S2, 69, 56	black , 48, random	0.00060, 0.817, 40 ,772
M74	qm9_4,8 , 240,A, full	S2, 257, 36	avg, 44, false	0.00184, 0.807, 44 ,434
M75	qm9_4,2 , 60,B, groups	S2, 81, 20	resize, 36,-	0.00119, 0.754, 36 ,521
M76	qm9_4,2 , 60,B, groups	S2, 31, 24	avg, 40, random	0.00084, 0.762, 16 ,723
M77	qm9_4,2 , 60,A, none	S2, 60, 44	avg, 40, random	0.00102, 0.799, 44 ,662
M78	qm9_2,2 , 120,D, groups	S2, 98, 44	resize, 32,-	0.00241, 0.828, 24 ,562

ID	DB, Number of images per molecule, training set size[10^3], image generation type , shuffling type	Architecture, Epochs, Batch size	Margins (type, size and orientation)	LR, Momentum, Patience, Accuracy of model mae, meV
M80	qm9_6,8 , 160,A, partial	S2, 55, 40	black , 36, random	0.00277, 0.829, 40 ,696
M81	qm9_6,4 , 80,A, groups	S2, 107, 20	avg, 48, random	0.00065, 0.816, 44 ,512
M82	qm9_4,8 , 240,D, partial	S2, 89, 24	avg, 36, random	0.00174, 0.84, 36 ,710
M84	qm9_4,8 , 240,A, full	S2, 54, 52	black , 44, random	0.00082, 0.817, 24 ,629
M85	qm9_3,1 , 40,D, groups	S2, 41, 44	black , 32, random	0.00258, 0.841, 20 ,765
M86	qm9_3,2 , 80,D, groups	S2, 67, 24	black , 48, random	0.00212, 0.825, 36 ,726
M88	qm9_4,4 , 120,A, groups	S2, 103, 40	resize, 48,-	0.00168, 0.754, 32 ,467
M90	qm9_4,8 , 240,A, none	S2, 96, 40	black , 40, random	0.00061, 0.833, 16 ,500
M91	qm9_3,8 , 320,D, groups	densenet121, 73, 16	black , 40, random	0.00150, 0.782, 44 ,648
M95	qm9,1 , 120,F, none	densenet121, 54, 52	avg, 36, random	0.00052, 0.826, 36 ,736
M96	qm9_2,4 , 240,A, partial	vgg19, 41, 44	avg, 36, random	0.00110, 0.818, 32 ,639
M98	qm9_3,4 , 160,B, groups	vgg19, 310, 56	resize, 48,-	0.00249, 0.825, 20 ,405
M99	qm9,2 , 240,E, groups	r34, 65, 24	resize, 40,-	0.00264, 0.828, 20 ,484
M100	qm9_6,8 , 160,E, groups	vgg16, 45, 56	avg, 32, random	0.00214, 0.796, 28 ,708
M101	qm9_4,4 , 120,E, groups	r50, 61, 32	avg, 40, random	0.00138, 0.774, 44 ,805
M102	qm9_3,2 , 80,C, partial	densenet121, 49, 20	resize, 40,-	0.00279, 0.775, 32 ,697
M105	qm9_2,1 , 60,D, none	vgg19, 334, 40	resize, 36,-	0.00039, 0.795, 44 ,332
M108	qm9_2,2 , 120,D, none	S2, 151, 40	resize, 32,-	0.00034, 0.799, 24 ,400
M109	qm9,1 , 120,C, none	sqn11, 49, 32	black , 48, random	0.00035, 0.763, 20 ,747
M111	qm9_3,8 , 320,F, partial	sqn10, 62, 24	resize, 32,-	0.00072, 0.842, 40 ,713
M112	qm9_3,8 , 320,E, partial	densenet121, 60, 52	black , 32, random	0.00159, 0.756, 44 ,761
M114	qm9,1 , 120,B, groups	sqn10, 98, 44	black , 40, random	0.00176, 0.816, 24 ,688
M115	qm9_2,2 , 240,E, partial	S2, 263, 20	avg, 32, false	0.00187, 0.829, 40 ,622
M116	qm9_4,2 , 60,D, none	vgg16, 37, 28	black , 36, random	0.00098, 0.85, 20 ,656

Table S22: Type of all trained models for prediction of bandgap (for second stage of research)

ID	DB, Number of images per molecule, training set size[10^3], image generation type , shuffling type	Architecture, Epochs, Batch size	Margins (type, size and orientation)	LR, Momentum, Patience, Accuracy of model mae, meV
M118	qm9_3,4 , 160,A, groups	S2, 151, 28	resize, 48,-	0.00152, 0.836, 32 ,379
M119	qm9_3,4 , 160,B, groups	vgg19, 225, 48	resize, 32,-	0.00092, 0.811, 20 ,468
M120	qm9_3,8 , 320,A, groups	S2, 167, 60	resize, 40,-	0.00221, 0.755, 40 ,345
M121	qm9_3,4 , 160,B, groups	vgg19, 320, 44	resize, 40,-	0.00121, 0.791, 24 ,412
M122	qm9,4 , 480,A, groups	S2, 146, 52	resize, 36,-	0.00294, 0.806, 36 ,334
M123	qm9_2,8 , 480,A, groups	S2, 83, 44	resize, 48,-	0.00200, 0.753, 16 ,324
M124	qm9_3,12 , 480,B, groups	vgg19, 97, 60	resize, 36,-	0.00072, 0.760, 28 ,402
M125	qm9,4 , 480,A, groups	S2, 142, 108	resize, 32,-	0.00084, 0.761, 24 ,341
M126	qm9_3,8 , 320,B, groups	vgg19, 78, 28	resize, 40,-	0.00189, 0.758, 32 ,454
M127	qm9_6,8 , 160,A, groups	S2, 104, 100	resize, 48,-	0.00105, 0.798, 28 ,372
M128	qm9,4 , 480,A, groups	S2, 79, 40	resize, 40,-	0.00199, 0.784, 36 ,373
M129	qm9,1 , 120,B, groups	vgg19, 428, 40	resize, 40,-	0.00150, 0.784, 28 ,420
M131	qm9_6,16 , 320,B, groups	vgg19, 216, 100	resize, 40,-	0.00274, 0.800, 28 ,399
M132	qm9,1 , 160,B, groups	vgg19, 70, 40	resize, 36,-	0.00109, 0.772, 20 ,463
M133	qm9_4,1 , 30,A, groups	S2, 51, 56	resize, 36,-	0.00182, 0.753, 24 ,541
M134	qm9_4,1 , 30,B, groups	vgg19, 188, 92	resize, 40,-	0.00145, 0.836, 28 ,540
M135	qm9_6,4 , 80,B, groups	vgg19, 214, 100	resize, 32,-	0.00031, 0.841, 16 ,504
M136	qm9_4,4 , 120,B, groups	vgg19, 157, 60	resize, 44,-	0.00123, 0.843, 32 ,421
M137	qm9_4,1 , 30,B, groups	vgg19, 109, 20	resize, 36,-	0.00140, 0.834, 28 ,542
M138	qm9_4,2 , 60,A, groups	S2, 119, 88	resize, 32,-	0.00034, 0.797, 32 ,505
M139	qm9_2,2 , 120,B, groups	vgg19, 129, 108	resize, 48,-	0.00100, 0.775, 28 ,406
M140	qm9_3,1 , 40,A, groups	S2, 67, 64	resize, 36,-	0.00124, 0.753, 36 ,574
M141	qm9,1 , 120,A, groups	S2, 94, 44	resize, 36,-	0.00264, 0.840, 24 ,416
M143	qm9,1 , 120,B, groups	vgg19, 196, 124	resize, 44,-	0.00227, 0.821, 44 ,403
M144	qm9,1 , 120,B, groups	vgg19, 116, 112	resize, 36,-	0.00251, 0.777, 32 ,452
M145	qm9_4,2 , 60,B, groups	vgg19, 104, 76	resize, 40,-	0.00223, 0.836, 24 ,461
M146	qm9_4,4 , 120,A, groups	S2, 117, 40	resize, 32,-	0.00213, 0.777, 32 ,409
M147	qm9_4,2 , 60,B, groups	vgg19, 78, 120	resize, 48,-	0.00246, 0.768, 16 ,456
M148	qm9_2,2 , 120,B, groups	vgg19, 236, 48	resize, 44,-	0.00195, 0.800, 44 ,426
M149	qm9_4,1 , 30,A, groups	S2, 63, 56	resize, 36,-	0.00035, 0.847, 24 ,610
M150	qm9_6,4 , 80,A, groups	S2, 122, 96	resize, 32,-	0.00041, 0.800, 24 ,541
M151	qm9_2,1 , 60,A, groups	S2, 91, 64	resize, 36,-	0.00085, 0.788, 32 ,489
M152	qm9_6,1 , 20,B, groups	vgg19, 72, 76	resize, 32,-	0.00299, 0.828, 16 ,619
M153	qm9_6,4 , 80,A, groups	S2, 187, 40	resize, 40,-	0.00134, 0.785, 44 ,408
M154	qm9_2,1 , 60,A, groups	S2, 69, 92	resize, 48,-	0.00130, 0.819, 24 ,435
M155	qm9_6,2 , 40,B, groups	vgg19, 43, 104	resize, 40,-	0.00162, 0.757, 16 ,547
M157	qm9_3,2 , 80,A, groups	S2, 73, 68	resize, 48,-	0.00046, 0.751, 36 ,454
M158	qm9_3,2 , 80,A, groups	S2, 243, 88	resize, 36,-	0.00295, 0.753, 32 ,424
M159	qm9_3,1 , 40,B, groups	vgg19, 77, 36	resize, 32,-	0.00253, 0.813, 28 ,543
M160	qm9_3,2 , 80,A, groups	S2, 81, 56	resize, 36,-	0.00086, 0.807, 36 ,437
M161	qm9_2,1 , 60,B, groups	vgg19, 236, 112	resize, 40,-	0.00124, 0.807, 44 ,458
M162	qm9_6,1 , 20,A, groups	S2, 70, 52	resize, 40,-	0.00151, 0.841, 36 ,632
M163	qm9_6,1 , 20,A, groups	S2, 86, 68	resize, 32,-	0.00088, 0.835, 44 ,664
M164	qm9_3,2 , 80,B, groups	vgg19, 91, 112	resize, 40,-	0.00176, 0.760, 28 ,449
M165	qm9_6,2 , 40,A, groups	S2, 93, 40	resize, 48,-	0.00068, 0.824, 24 ,528
M166	qm9_6,2 , 40,A, groups	S2, 94, 124	resize, 44,-	0.00200, 0.770, 40 ,521
M167	qm9_2,1 , 60,A, groups	S2, 94, 96	resize, 40,-	0.00202, 0.827, 24 ,480
M168	qm9_6,2 , 40,B, groups	vgg19, 195, 92	resize, 36,-	0.00132, 0.832, 44 ,518
M169	qm9_6,16 , 320,A, groups	S2, 288, 92	resize, 36,-	0.00198, 0.844, 40 ,369

ID	DB, Number of images per molecule, training set size[10^3], image generation type , shuffling type	Architecture, Epochs, Batch size	Margins (type, size and orientation)	LR, Momentum, Patience, Accuracy of model mae, meV
M170	qm9_3,12 , 480,A, groups	S2, 174, 24	resize, 48,-	0.00042, 0.752, 40 ,324
M171	qm9_6,2 , 40,A, groups	S2, 71, 28	resize, 32,-	0.00038, 0.798, 32 ,617
M172	qm9,2 , 240,B, groups	vgg19, 74, 84	resize, 48,-	0.00295, 0.786, 16 ,396
M173	qm9_2,4 , 240,B, groups	vgg19, 181, 108	resize, 40,-	0.00158, 0.803, 40 ,393
M174	qm9_6,1 , 20,A, groups	S2, 62, 112	resize, 48,-	0.00232, 0.827, 32 ,627
M175	qm9_6,2 , 40,A, groups	S2, 89, 44	resize, 44,-	0.00065, 0.815, 44 ,550
M176	qm9_6,2 , 40,A, groups	S2, 59, 76	resize, 32,-	0.00161, 0.819, 24 ,597
M177	qm9_2,4 , 240,B, groups	vgg19, 90, 96	resize, 40,-	0.00135, 0.789, 16 ,403
M178	qm9_2,1 , 60,B, groups	vgg19, 109, 92	resize, 44,-	0.00175, 0.827, 40 ,469
M179	qm9_3,1 , 40,B, groups	vgg19, 81, 80	resize, 40,-	0.00274, 0.756, 36 ,527
M180	qm9_3,2 , 80,A, groups	S2, 73, 64	resize, 48,-	0.00081, 0.840, 32 ,426
M181	qm9_4,16 , 480,A, groups	S2, 289, 112	resize, 32,-	0.00062, 0.839, 24 ,350
M182	qm9_3,1 , 40,B, groups	vgg19, 154, 88	resize, 48,-	0.00124, 0.844, 36 ,491
M183	qm9_6,2 , 40,A, groups	S2, 68, 24	resize, 44,-	0.00116, 0.785, 36 ,504
M184	qm9_3,1 , 40,B, groups	vgg19, 90, 20	resize, 44,-	0.00167, 0.752, 20 ,504
M185	qm9_2,1 , 60,A, groups	S2, 71, 44	resize, 44,-	0.00031, 0.842, 28 ,462
M186	qm9_2,1 , 60,A, groups	S2, 55, 72	resize, 44,-	0.00179, 0.836, 16 ,512
M187	qm9_3,1 , 40,B, groups	vgg19, 154, 76	resize, 32,-	0.00296, 0.762, 36 ,525
M188	qm9_3,2 , 80,A, groups	S2, 72, 48	resize, 48,-	0.00051, 0.847, 20 ,424
M189	qm9_6,2 , 40,B, groups	vgg19, 99, 40	resize, 40,-	0.00253, 0.834, 24 ,497
